# A Hybrid Ecological Momentary Compassion–Focused Intervention for Enhancing Resilience in Help-Seeking Young People: Prospective Study of Baseline Characteristics in the EMIcompass Trial

**DOI:** 10.2196/39511

**Published:** 2022-11-04

**Authors:** Isabell Paetzold, Anita Schick, Christian Rauschenberg, Dusan Hirjak, Tobias Banaschewski, Andreas Meyer-Lindenberg, Sebastian Butz, Chiara Floesser, Leonie Schueltke, Jan Rasmus Boehnke, Benjamin Boecking, Ulrich Reininghaus

**Affiliations:** 1 Department of Public Mental Health Central Institute of Mental Health Medical Faculty Mannheim, Heidelberg University Mannheim Germany; 2 Department of Psychiatry and Psychotherapy Central Institute of Mental Health Medical Faculty Mannheim, Heidelberg University Mannheim Germany; 3 Department of Child and Adolescent Psychiatry and Psychotherapy Central Institute of Mental Health Medical Faculty Mannheim, Heidelberg University Mannheim Germany; 4 School of Health Sciences University of Dundee Dundee United Kingdom; 5 Tinnitus Center Charité Universitätsmedizin Berlin Berlin Germany; 6 ESRC Centre for Society and Mental Health and Social Epidemiology Research Group King's College London London United Kingdom; 7 Health Service and Population Research Department Centre for Epidemiology and Public Health Institute of Psychiatry, Psychology & Neuroscience, King's College London London United Kingdom

**Keywords:** mobile health intervention, mHealth intervention, digital intervention, just-in-time adaptive intervention, JITAI, blended care, public mental health, inclusiveness, transdiagnostic, clinical staging, intervention manual, mobile phone

## Abstract

**Background:**

Young people are a target population for mental health–related early intervention and prevention. Although evidence for early intervention is promising, availability of and access to youth mental health services remain limited. Therefore, the development of an evidence-based hybrid intervention is urgently needed.

**Objective:**

This study aimed to present a manual for a hybrid intervention, combining an ecological momentary intervention and face-to-face sessions aimed for enhancing resilience in help-seeking young people based on compassion-focused interventions, and explore whether participants’ baseline characteristics are associated with putative mechanisms and outcomes of the EMIcompass intervention. Specifically, we aimed to explore initial signals as to whether participants’ sociodemographic, clinical, and functional characteristics at baseline are associated with putative mechanisms (ie, change in self-compassion, change in emotion regulation, working alliance, training frequency); and whether participants’ sociodemographic, clinical, and functional characteristics, self-compassion, and emotion regulation at baseline are associated with clinical outcomes (ie, psychological distress and general psychopathology at postintervention and 4-week follow-ups) in the experimental condition and obtain first parameter estimates.

**Methods:**

We recruited young people aged 14 to 25 years, with psychological distress, Clinical High At-Risk Mental State, or first episodes of severe mental disorder for an exploratory randomized controlled trial with assessments at baseline and postintervention and 4-week follow-ups. A structured manual was developed and optimized based on a pilot study’s manual, a scoping review of existing literature and manuals, exchange with experts, the team’s clinical experience of working with compassion-focused interventions, and the principles of ecological momentary interventions. This analysis focuses on the experimental condition receiving the EMIcompass intervention.

**Results:**

A total of 46 young individuals were randomized to the experimental condition. There was evidence for initial signals of effects of age (B=0.11, 95% CI 0.00-0.22), general psychopathology (B=0.08, 95% CI −0.01 to 0.16), and clinical stage (B=1.50, 95% CI 0.06-2.93) on change in momentary self-compassion and change in emotion regulation from baseline to postintervention assessments. There was no evidence for associations of other baseline characteristics (eg, gender, minority status, and level of functioning) and putative mechanisms (eg, overall self-compassion, working alliance, and training frequency). In addition, except for an initial signal for an association of momentary self-compassion at baseline and psychological distress (B=−2.83, 95% CI −5.66 to 0.00), we found no evidence that baseline characteristics related to clinical outcomes.

**Conclusions:**

The findings indicated the reach of participants by the intervention largely independent of sociodemographic, clinical, and functional baseline characteristics. The findings need to be confirmed in a definitive trial.

**Trial Registration:**

German Clinical Trials Register NDRKS00017265; https://www.drks.de/drks_web/navigate.do?navigationId=trial.HTML&TRIAL_ID=DRKS00017265

**International Registered Report Identifier (IRRID):**

RR2-10.2196/27462

## Introduction

### Background

Young people constitute a priority target population for mental health–related prevention and early intervention, as they are particularly affected by mental health problems. Mental disorders primarily emerge in adolescence and young adulthood, and >60% of all lifetime cases have their onset before the age of 25 years [1]. With a worldwide pooled prevalence of 21% of mental disorders in adolescents aged 12 to 18 years [2], mental health problems contribute substantially to the disease burden [3,4]. Addressing the co-occurrence and overlap of subclinical and clinical experiences and symptoms [5-8], especially in the early stages of psychopathology, dimensional classification frameworks [9,10] cutting across traditional diagnostic boundaries, including the Hierarchical Taxonomy of Psychopathology (HiTOP) [11], have been proposed. Clinical staging models take early, overlapping, and nonspecific psychopathological symptoms and transitional staging processes into account [12,13].

There is convincing evidence on risk factors that are modifiable, on mental health problems that can be changed, and on protective factors that can be strengthened to enhance resilience [14-16]. Traditional psychotherapeutic interventions, including standard cognitive behavioral therapy, as well as third-wave approaches, show moderate to high effect sizes in randomized controlled trials (RCTs) and meta-analyses [17-20]. However, there is considerable room for improvement, as—even after successful treatment—many service users show significant residual symptoms or relapse [21]. In addition, the availability of and access to youth mental health services remain limited [22,23]. More downstream, this may result in a longer duration of untreated illness, an important marker of poor prognosis and complex course and outcome [24,25].

Some of these problems of standard care might be caused by difficulties transferring preventive and therapeutic strategies developed in face-to face sessions to service users’ daily life. Mobile health (mHealth) may be a promising approach to address these challenges by improving access to mental health care for young people by using mobile devices for the delivery of prevention and intervention [26-30]. With ecological momentary assessment (EMA), often also referred to as experience sampling methodology (ESM) [26,31], a structured diary method, momentary fluctuations in experience and behavior can be assessed in real time and real life. Ecological momentary interventions (EMIs) [26,29,32-34] offer the opportunity to deliver adaptive and personalized intervention components in daily life. The digital approach may help to lower the threshold for young people to access interventions meeting their needs and preferences and facilitates the ecological translation of techniques learned into service users’ everyday lives [29]. A recent nationally representative survey indicated that young people do frequently use mHealth apps and are even more likely to do so when feeling distressed [30].

However, digital approaches are also confronted with challenges: most apps currently available in major app stores are not evidence based, and some even include potentially harmful content [28,35]. In addition, the reach of digital interventions has been subject to controversial debate, as concerns have been expressed that barriers to treatment may be created rather than removed [36,37]. A review indicated that studies of the effectiveness of mHealth apps mostly include samples of predominantly female, White participants with an average age of 30 to 45 years [38], and the degree of generalizability of findings to service users with other characteristics remains largely unexplored. Therefore, the development of evidence-based, low-threshold interventions that specifically target established candidate mechanisms that have been linked to the development and persistence of mental health conditions across various groups and settings is urgently needed. In addition, it is crucial to explore the association of participants’ baseline characteristics with putative mechanisms and outcomes to examine the reach of the intervention.

Extensive research identified stress reactivity as a putative transdiagnostic mechanism in the development of psychopathology and a promising target for prevention and early intervention [26]. Stress reactivity (ie, increases in negative affect in response to minor daily stressors) is thought to be a behavioral marker of stress sensitization, positing that frequent or chronic experiences of adversity may gradually increase individuals’ stress response to subsequent adversities and minor stressors in everyday life [26,39,40].

Compassion-focused interventions (CFIs) may be a promising approach to target stress reactivity in daily life. Building on a combination of evolutionary psychology, attachment theory, and social mentality theory, the compassion-focused approach claims that various psychological problems are caused by unhelpful loops among distressing emotions, defensive behaviors, and cognitive processes such as rumination, worry, and self-criticism [41]. A model with 3 interrelated major emotional systems is suggested [41-43]: threat, drive, and soothing. Many people experience an overactive threat system, an overactive or somehow blocked drive system, and an underactive soothing system [41]. Therefore, CFIs focus on strengthening the soothing system, as it is thought to be an antagonist to an overactive threat system and a good basis for a well-functioning drive system. CFIs are not symptom specific, and previous studies demonstrated that they are an effective treatment for various mental health problems [19,44-46]. Positive imagery, a key component of CFIs, has been shown to effectively reduce a wide range of mental health problems and increases positive affect, optimism, and behavioral activation [46-50]. In laboratory studies, the application of compassion-focused techniques has been shown to reduce state negative affect and paranoia in moments of high stress [49,51].

Combining digital approaches and CFIs in a hybrid intervention using imagery-based techniques may be particularly well-suited to target stress reactivity in the daily life of young people. Previous research indicated higher acceptability and larger effect sizes for hybrid interventions in comparison with stand-alone internet- and mobile-based interventions [52,53]. Therefore, EMIcompass was developed as a hybrid intervention combining an EMI with guided face-to-face sessions. A pilot study provided initial evidence for feasibility, safety, and beneficial effects of a compassion-focused EMI for enhancing resilience in help-seeking young people [54]. Feasibility and initial signals of efficacy of the intervention have been investigated in a registered exploratory RCT in Germany [55], comparing treatment as usual (TAU) with TAU+EMIcompass in young people with early mental health problems.

### Objectives

This paper aims to (1) present the intervention manual for EMIcompass, a hybrid intervention combining an EMI and face-to-face sessions aiming at enhancing resilience in help-seeking young people based on compassion-focused principles [41-43] and (2) explore whether participants’ baseline characteristics are associated with putative mechanisms and outcomes of the EMIcompass intervention. To this end, we aimed to obtain first parameter estimates and explore initial signals as to whether sociodemographic, clinical, and functional characteristics at baseline (ie, clinical stage, psychological distress, general psychopathology, level of functioning, age, gender, and minority status) are associated with putative mechanisms (ie, change in self-compassion, change in emotion regulation, working alliance, and training frequency); and sociodemographic, clinical, and functional characteristics (ie, clinical stage, psychological distress, general psychopathology, level of functioning, age, gender, and minority status) as well as self-compassion and emotion regulation at baseline are associated with clinical outcomes (ie, psychological distress and general psychopathology at postintervention and 4-week follow-ups) in the experimental condition and obtain 95% CIs.

## Methods

### Study Design

In our exploratory RCT, participants were randomly allocated to a control condition of TAU or an experimental condition of TAU+EMIcompass in a 50:50 ratio. For this analysis, data from the experimental condition were used to examine the impact of participants’ baseline characteristics on the putative mechanisms and outcomes of the intervention. In the RCT, candidate mechanisms (primary: stress reactivity; secondary: resilience, interpersonal sensitivity, threat anticipation, and negative affective appraisals) and outcomes (primary: psychological distress; secondary: primary psychiatric symptoms, general psychopathology, and quality of life) were assessed at baseline (ie, before randomization), at the end of the intervention, and at the 4-week follow-up. Observer ratings were performed by blinded assessors. The sample size was based on a power simulation for the primary outcome of the trial [56]. The RCT was conducted between August 2019 and September 2021. Appointments were held in person or via video calls (owing to the COVID-19 pandemic). Further details on study procedures are described in the study protocol [56].

### Ethics Approval

The trial has received ethical approval from the local ethics committee of the Medical Faculty Mannheim, Heidelberg University (2017-602N-MA). All participants and, in case of minors, parents or legal guardians, provided written informed consent before inclusion in the study.

### Manual for the EMIcompass Intervention

To ensure consistent delivery of the intervention, a structured manual was developed and refined building on the manual from the pilot study (Multimedia Appendix 1 provides changes to the pilot version) [54]. The development and optimization process comprised a scoping review of available literature and existing manuals. In addition, local CFI experts were consulted, and the team’s clinical experience of working with these approaches was considered. The intervention was designed based on principles of EMIs [26,27,29,34].

**Figure 1:**
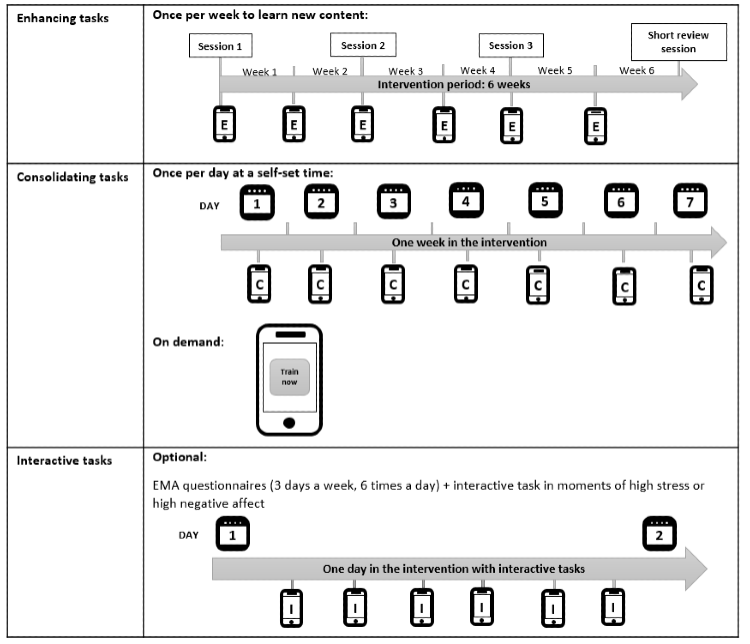
Overview of the intervention structure and the types of tasks. EMA: ecological momentary assessment.

**Figure 2:**
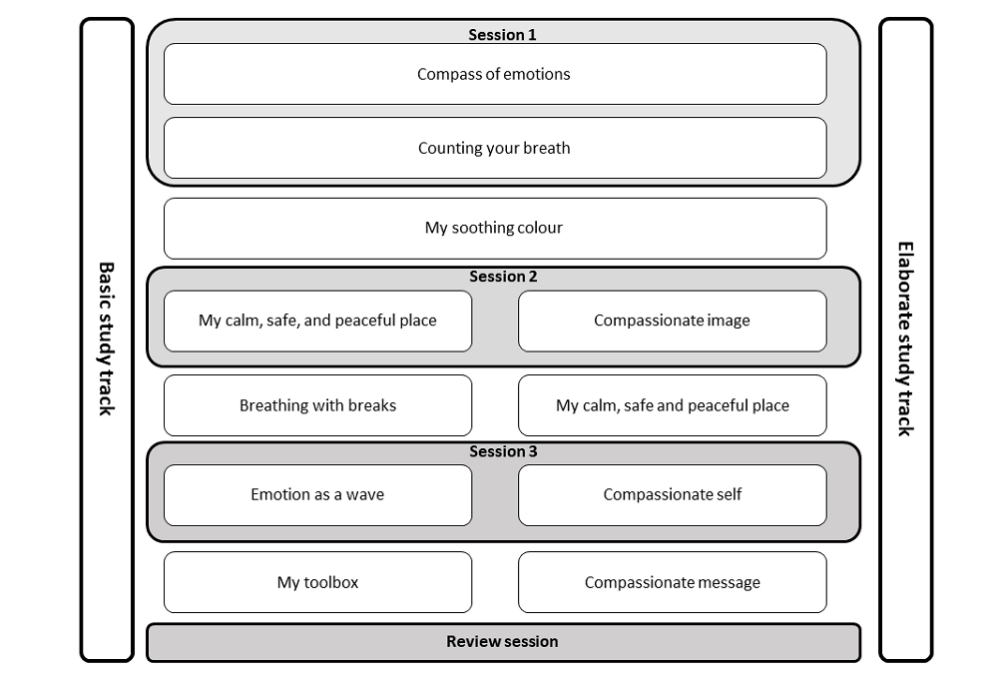
Summary of the intervention content.

The development and optimization process resulted in a structured manual for a 6-week intervention combining 4 individual sessions with daily training via a dedicated smartphone app. The manual is reported in the Multimedia Appendix 2 in line with state-of-the art guidelines such as World Health Organization guidelines for reporting health interventions using mobile phones [57] as well as the Template for Intervention Description and Replication Checklist [58]. An overview of the intervention structure and the types of tasks is provided in Figure 1. Figure 2 displays a summary of the intervention content. The intervention can be aligned to participants’ personal needs; for example, sessions or training weeks can be repeated if necessary. Moreover, the intervention provides 2 different study tracks with varying foci and demand levels. On the basis of the trained psychologists’ impression and the participants’ experiences in the first 2 weeks of the intervention, participants were allocated to the basic or the elaborate track of the intervention. The basic study track focused on creating feelings of safeness and calmness by introducing breathing techniques and soothing imagery. The elaborate track extended breathing exercises and soothing imagery by introducing self-compassionate imagery and writing.

The intervention comprised 3 guided sessions to introduce compassion-focused principles and practical tasks to activate participants’ soothing system and to provide feedback on their current progress and a short review session. The content was presented on the smartphone and was discussed with the trained psychologist. All sessions could be delivered in person or via video calls. The in-person sessions were delivered in dedicated treatment and assessment rooms. For sessions delivered via video call, participants attended the sessions at home. Psychologists were trained in delivering the EMIcompass intervention and supervised by an expert in CFIs (BB) to ensure intervention quality.

To facilitate real-time and real-world translation of techniques into participants’ daily lives, an EMI was administered through an mHealth app (movisensXS) on a study smartphone that they received in the first guided session. To learn new techniques, participants were asked to complete one *enhancing* task per week, which was subsequently extended over the intervention. In the weeks with sessions, the new task was introduced in contact with the trained psychologists; in the weeks without session, participants familiarized with the new enhancing task autonomously. Short *consolidating* tasks were offered to practice the techniques previously introduced in enhancing tasks. Once a day, at a time set by the participants, a signal was prompted to offer participants a consolidating task. In addition, on-demand consolidating tasks were available at any time during the intervention. Furthermore, participants could decide whether they also wanted to allow for *interactive* tasks. To present interactive tasks, the smartphone prompted a signal 6 times per day on 3 consecutive days per week at random within set blocks of time. At each signal, participants were asked to complete a short EMA questionnaire on momentary stress and affect. If participants indicated high stress or negative affect in the EMA, they were offered an interactive task. Thereby, the interactive tasks guided participants to use previously learned compassion-focused techniques in moments of distress, which is an essential element of CFIs [42]. A gamification element was used to provide feedback on the progress made. If appropriate, participants could choose between reading the instructions on the smartphone’s screen and a guided audio version of the tasks.

Between sessions, participants received weekly feedback on their progress and were offered email and phone contact to discuss questions and technical problems. At the beginning of weeks without scheduled session (ie, weeks 2, 4, and 6), participants were contacted to notify them about a new enhancing task becoming available for them to try out autonomously. To proceed with the subsequent study week, participants had to complete at least one consolidating task per week. If this was not the case, the intervention week was repeated.

### Participants

In line with a modified version of the clinical staging model [12,56], the EMIcompass study recruited young individuals aged 14 to 25 with current psychological distress, Clinical High At-Risk Mental State (CHARMS), or a first treated episode of severe mental disorder (for a detailed description of the modified criteria, see Multimedia Appendix 3 [12,56,59-64]; age range based on suggestions of the youth mental health reform and local regulations [65]). Participants were recruited from mental health services at the Central Institute of Mental Health, Mannheim, Germany, via local registries and advertisements on the institute’s webpage and social media. Self-reported and observer-rated measures were used to assess eligibility to participate. All participants (including caregivers for minors) provided informed consent and were reimbursed for their time and travel expenses. Further details on inclusion and exclusion criteria are provided in the study protocol [56].

### Measures

Multimedia Appendix 4 [12,59,60,66-78] provides an overview of the measures used and the time points of administration. We used self-reports and, in the case of ethnicity, family assessments to collect data on sociodemographic characteristics. Clinical characteristics (ie, clinical stage, psychological distress, general psychopathology, and level of functioning) were assessed using self-report questionnaires, observer ratings, and standardized interviews. Self-report questionnaires were used to assess overall self-compassion, emotion regulation, and working alliance. Momentary self-compassion was assessed using EMA. The total number of training tasks completed in the EMI was used as an indicator of training frequency. Multimedia Appendix 5 displays a correlation table of the measures used.

### Statistical Analysis

The study was registered on the open science framework prior to accessing the data [79]. To obtain parameter estimates for the effect of sociodemographic, clinical, and functional characteristics on putative mechanisms and processes, we fitted linear regression models with change in self-compassion (δ_postintervention−baseline_), change in adaptive and maladaptive emotion regulation (δ_postintervention−baseline_), working alliance (patient and therapist ratings and total scores), and training frequency (total score) as dependent variables. Independent variables in the models were clinical stage (stage 1a, stage 1b, and stage 2), psychological distress, general psychopathology, level of functioning, age, gender (female and male), and ethnic minority status (minority and majority). Parameter estimates (95% CIs) were obtained for the main effects of baseline characteristics on change in self-compassion, change in adaptive and maladaptive emotion regulation, working alliance, and training frequency. We computed partial η^2^ as estimators of effect size for the predictors.

To obtain parameter estimates for the effect of sociodemographic, clinical, and functional characteristics and baseline level of self-compassion, adaptive, and maladaptive emotion regulation on clinical outcomes, we fitted mixed effects regression models with psychological distress and general psychopathology at postintervention or at follow-up as the dependent variables. Independent variables in these models were time (postintervention and follow-up), clinical stage (stage 1a, stage 1b, and stage 2), level of functioning at baseline, age, gender (female and male), ethnic minority status (minority and majority), momentary and overall self-rated self-compassion at baseline, adaptive and maladaptive emotion regulation at baseline, psychological distress at baseline (as independent variable in the model with general psychopathology at postintervention or follow-up as outcome and as control variable with psychological distress at postintervention or follow-up as outcome), and general psychopathology at baseline (as independent variable in the model with psychological distress at postintervention or follow-up as outcome and as control variable with general psychopathology at postintervention or follow-up as outcome). We took into account the within-subject clustering of repeated measures by adding a level-2 random intercept. The model was fitted using restricted maximum likelihood estimation. Parameter estimates (95% CIs) were obtained for the main effects of baseline characteristics on outcomes across the 2 follow-up (ie, postintervention and 4-week follow-ups). In the next step, given the exploratory nature of this trial, 95% CIs for the 2 time-specific contrasts were obtained. For this, the aforementioned model was extended by time×characteristic interactions (time×clinical stage, time×psychological distress, time×general psychopathology, time×level of functioning, time×age, time×gender, time×self-compassion, time×adaptive emotion regulation, and time×maladaptive emotion regulation). The “margins” command was used for each interaction to obtain predicted means for both time points and all manifestations of categorical variables (eg, “margins time point #clinical stage”). For continuous variables, the “margins” command was used with z-standardized continuous variables to obtain predicted means for both time points and low (mean−1 SD), mean, and high (mean+1 SD) levels of the given continuous variable (eg, “margins, at [z_age = (−1 0 1)] over [time]”).

To transform the results into an effect size, the model was run including only a random intercept for participants, the estimated target relationship, and the baseline control to obtain the conditional and pooled variance across both assessment time points [78,80,81]. The resulting estimate of variance therefore approximates the variation in the dependent variable at any cross-section in postintervention and follow-up. The resulting estimate is on a similar scale as other typical d-type effect sizes (at “0” of any random slopes, if included), and if additional random effects were strong, these variances are underestimations, and the effect sizes in the following likely are at the upper possible limit.

The analysis was conducted according to intention-to-treat principles, with data from all participants entered into the analysis, including those who have low adherence to or who dropped out of the intervention. To screen for potential collinearity problems, we computed variance inflation factors and tolerance values (Multimedia Appendix 6).

## Results

### Basic Sample and Clinical Characteristics

An overview of basic sample and clinical characteristics is displayed in Table 1. The sample of those randomized to the experimental condition comprised 46 individuals (50% of the total sample in the exploratory RCT of N=92), with a mean age of 21.30 (SD 2.84; range 14-25) years. Most participants (35/46, 76%) identified as girls or women, 24% (11/46) of the participants identified as boys or men, and no participant identified as nonbinary. We identified 70% (32/46) of participants as White majority (German), 9% (4/46) as White other, and 22% (10/46) as other or mixed ethnicity. Most participants were classified as stage 1a (psychological distress, 26/46, 57%), 28% (13/46) of the participants met criteria for stage 1b (CHARMS), and 15% (7/46) of the participants were classified as stage 2 (first episode of severe mental disorder). The mean level of psychological distress at baseline was 28.20 (SD 5.08), and the mean level of general psychopathology at baseline was 24.55 (SD 9.94). The average level of functioning was 71.83 (SD 9.89). Participants showed comparable levels of overall self-rated self-compassion (*P*=.33) and adaptive (*P*=.57) and maladaptive emotion regulation (*P*=.21) at baseline and postintervention. We observed increases in momentary self-compassion at postintervention (*P*=.02).

**Table 1:** Basic sample and clinical characteristics.

					Baseline (n_max_=46)^a^			Postintervention (n_max_=45)				Follow-up (n_max_=45)				Baseline v postintervention					
																*t* test (*df*)				*P* value	
Age at baseline (years), mean (SD)					21.30 (2.84)			—^b^				—				—				—	
**Gender, n (%)**									—				—				—				—
	Female			35 (76)																	
	Male			11 (24)																	
	Nonbinary			0 (0)																	
**Ethnicity, n (%)**									—				—				—				—
	White majority			32 (70)																	
	**Minority**																				
		Mixed White majority or White other	3 (7)																		
		White other	4 (9)																		
		Turkish	3 (6)																		
		Mixed other	2 (4)																		
		Middle East	1 (2)																		
		Asian	1 (2)																		
**Level of education, n (%)**									—				—				—				—
	School: General Certificate of Secondary Education			7 (15)																	
	Further: A levels			14 (30)																	
	Higher: university			25 (54)																	
**Employment status, n (%)**									—				—				—				—
	Student			39 (85)																	
	School			4 (9)																	
	Vocational training or university			35 (76)																	
	Employed			4 (9)																	
	Unemployed			3 (6)																	
**Clinical stage at baseline, n (%)**									—				—				—				—
	1a			26 (57)																	
	1b			13 (28)																	
	2			7 (15)																	
Level of functioning at baseline, mean (SD)					71.83 (9.89)			—				—				—				—	
Psychological distress, mean (SD)					28.20 (5.08)			24.11 (6.55)				22.73 (7.16)				—				—	
General psychopathology, mean (SD)					24.55 (9.94)			18.0 (12.03)				16.20 (10.68)				—				—	
**Self-compassion,** **mean (SD)**																					
	Overall self-rating			18.34 (2.77)			18.70 (2.06)				—				−0.99 (42)				.33		
	Momentary rating			3.89 (0.87)			4.30 (1.06)				—				−2.35 (44)				.02		
**Emotion regulation, mean (SD)**																					
	Adaptive			5.51 (1.45)			5.61 (1.57)				—				−0.57 (42)				.57		
	Maladaptive			5.97 (1.46)			5.64 (1.45)				—				1.27 (42)				.21		
Training frequency, mean (SD)					—			75.84 (85.09)				—				—				—	
**Working alliance, mean (SD)**																					
	Patient rating			—			48.07 (8.37)				—				—				—		
	Therapist rating			—			46.74 (6.47)				—				—				—		

^a^Sample sizes varied owing to missing values at baseline (n_max_=46; n_min_=45).

^b^Not available.

### Sociodemographic, Clinical, and Functional Characteristics at Baseline Associated With Putative Mechanisms and Processes of Change

Table 2 presents the associations of sociodemographic, clinical, and functional characteristics at baseline with change in self-compassion and emotion regulation. There was no evidence for initial signals that participants’ characteristics at baseline were associated with change in overall self-rated self-compassion (δ_postintervention−baseline_)_._ For change in momentary self-compassion, we observed a tendency for an association with age (B=0.11, 95% CI 0.00-0.22): older participants tended to show more pronounced change in momentary self-compassion from baseline to postintervention. Clinical stage was associated with change in adaptive emotion regulation such that participants in stage 2 showed more pronounced positive changes in adaptive emotion regulation compared with participants in stage 1a (B=1.50, 95% CI 0.06-2.93). For change in maladaptive emotion regulation, we found a tendency for an association with general psychopathology such that participants with lower levels of psychopathology at baseline tended to show more pronounced reductions in maladaptive emotion regulation (B=0.08, 95% CI −0.01 to 0.16).

Table 3 presents the associations of sociodemographic, clinical, and functional characteristics at baseline with working alliance and training frequency. We found no evidence for initial signals of associations of working alliance and training frequency with baseline characteristics.

**Table 2:** Associations of sociodemographic, clinical, and functional characteristics at baseline with change in self-compassion and emotion regulation.

			Putative mechanisms of change														
			Change in overall self-rated self-compassion (n=43)				Change in momentary self-compassion (n=45)				Change in adaptive emotion regulation (n=43)				Change in maladaptive emotion regulation (n=43)		
			B (95% CI)		Effect size^a^		B (95% CI)		Effect size		B (95% CI)		Effect size		B (95% CI)		Effect size
Age			−0.05 (−0.40 to 0.29)		0.00		0.11 (0.00 to 0.22)		0.10		−0.10 (−0.23 to 0.04)		0.05		−0.09 (−0.26 to 0.09)		0.03
Gender			0.81 (−1.68 to 3.29)		0.01		−0.07 (−0.85 to 0.71)		0.00		−0.70 (−1.70 to 0.30)		0.06		0.45 (−0.81 to 1.72)		0.02
Ethnic minority status			0.91 (−1.28 to 3.10)		0.02		−0.20 (−0.89 to 0.49)		0.01		0.07 (−0.81 to 0.95)		0.00		0.51 (−0.60 to 1.62)		0.02
**Clinical stage^b^**					0.01				0.04				0.15				0.00
	Stage 1b	0.57 (−1.66 to 2.81)				−0.31 (−1.03 to 0.40)				0.70 (−0.20 to 1.60)				0.18 (−0.95 to 1.32)			
	Stage 2	−0.34 (−3.91 to 3.23)				0.27 (−0.81 to 1.34)				1.50 (0.06 to 2.93)				0.13 (−1.68 to 1.94)			
Psychological distress			−0.01 (−0.38 to 0.35)		0.00		0.03 (−0.09 to 0.14)		0.01		−0.02 (−0.16 to 0.13)		0.00		−0.15 (−0.34 to 0.03)		0.08
General psychopathology			−0.01 (−0.18 to 0.16)		0.00		0.03 (−0.02 to 0.09)		0.04		−0.04 (−0.11 to 0.03)		0.03		0.08 (−0.01 to 0.16)		0.09
Level of functioning			−0.01 (−0.11 to 0.10)		0.00		−0.02 (−0.06 to 0.01)		0.04		0.00 (−0.04 to 0.05)		0.00		0.01 (−0.05 to 0.07)		0.00

^a^Effect size partial η^2^.

^b^Stage 1a (individuals with psychological distress) as reference category.

**Table 3:** Associations of sociodemographic, clinical, and functional characteristics at baseline with working alliance and training frequency.

			Putative mechanisms of change											
			Working alliance—patient rating (n=44)				Working alliance—therapist rating (n=43)					Training frequency (n=45)		
			B (95% CI)		Effect size^a^		B (95% CI)		Effect size		B (95% CI)			Effect size
Age			0.57 (−0.33 to 1.46)		0.05		0.17 (−0.59 to 0.94)		0.01		2.69 (−7.38 to 12.77)			0.01
Gender			2.55 (−3.93 to 9.03)		0.02		2.72 (−2.93 to 8.37)		0.03		12.80 (−85.02 to 9.43)			0.00
Ethnic minority status			1.89 (−3.65 to 7.44)		0.01		1.54 (−3.18 to 6.26)		0.01		−26.31 (88.48 to 35.86)			0.02
**Clinical stage^b^**					0.09				0.04					0.05
	Stage 1b	5.03 (−0.68 to 10.74)				−1.22 (−5.93 to 3.49)				26.07 (−38.45 to 90.60)				
	Stage 2	−0.41 (−9.47 to 8.65)				3.51 (−4.00 to 11.02)				−41.07 (−141.05 to 58.91)				
Psychological distress			0.70 (−0.24 to 1.65)		0.06		0.33 (−0.46 to 1.12)		0.02		4.83 (−5.80 to 15.47)			0.02
General psychopathology			0.00 (−0.44 to 0.44)		0.00		−0.13 (−0.49 to 0.23)		0.02		−1.82 (−6.74 to 3.11)			0.02
Level of functioning			0.01 (−0.28 To 0.29)		0.00		0.11 (−0.14 To 0.36)		0.02		−0.31 (−3.50 to 2.87)			0.00

^a^Effect size partial η^2^.

^b^Stage 1a (individuals with psychological distress) as reference category.

### Sociodemographic, Clinical, and Functional Characteristics; Self-compassion; and Emotion Regulation at Baseline Associated With Clinical Outcomes

Table 4 presents findings on associations of psychological distress with participants’ characteristics and level of putative mechanisms at baseline and predicted marginal means. There was some evidence for a main effect of momentary self-compassion such that higher momentary self-compassion at baseline tended to be associated with, on average, lower levels of psychological distress across postintervention and follow-up assessments (B=−2.83, 95% CI −5.66 to 0.00). There was no evidence for main effects of sociodemographic or clinical characteristics, overall self-rated self-compassion, and emotion regulation on psychological distress.

Table 5 presents findings on associations of general psychopathology with participants’ characteristics and level of putative mechanisms at baseline and predicted marginal means. There was no evidence for initial signals of main effects of sociodemographic, clinical, and functional characteristics on general psychopathology.

Cross-differences between high and low levels of baseline characteristics at the time points are presented in Multimedia Appendix 7 [82].

**Table 4:** Associations of psychological distress with participants’ characteristics and level of putative mechanisms and processes at baseline and predicted marginal means^a^.

		Postintervention		Follow-up			Adjusted B (95% CI)			Effect size^b^	
		Predicted marginal mean (95% CI)	SE	Predicted marginal mean (95% CI)	SE						
Time		N/A^c^	N/A	N/A	N/A	−7.46 (−37.20 to 22.29)			−1.16		
Age								−0.31 (−1.04 to 0.42)			−0.05
	Low^d^	24.92 (22.26 to 27.57)	1.36	24.99 (22.33 to 27.65)	1.36						
	Mean	24.03 (22.33 to 25.73)	0.87	22.62 (20.93 to 24.32)	0.87						
	High^e^	23.15 (20.44 to 25.86)	1.38	20.26 (17.55 to 22.96)	1.38						
**Gender**								−0.51 (−5.26 to 4.23)			−0.08
	Female	24.16 (22.15 to 26.17)	1.03	23.90 (21.89 to 25.91)	1.03						
	Male	23.65 (19.61 to 27.69)	2.06	18.45 (14.41 to 22.49)	2.06						
**Ethnic minority status**								2.39 (−4.40 to 9.18)			0.37
	White majority	23.83 (22.02 to 25.64)	0.92	22.71 (20.91 to 24.52)	0.92						
	Minority	26.22 (19.81 to 32.62)	3.27	22.11 (15.70 to 28.52)	3.27						
**Clinical stage^f^**								−0.19 (−3.62 to 3.24)			−0.03
	Stage 1a	25.42 (23.12 to 27.73)	1.18	23.75 (21.44 to 26.05)	1.18						
	Stage 1b	21.84 (18.58 to 25.10)	1.66	20.21 (16.95 to 23.46)	1.66						
	Stage 2	22.62 (16.44 to 28.79)	3.15	23.37 (17.19 to 29.55)	3.15						
**General psychopathology at baseline**								0.04 (−0.29 to 0.38)			0.01
	Low	23.62 (19.88 to 27.37)	1.91	18.79 (15.05 to 22.54)	1.91						
	Mean	24.03 (22.33 to 25.74)	0.87	22.56 (20.86 to 24.26)	0.87						
	High	24.45 (20.85 to 28.04)	1.83	26.33 (22.73 to 29.92)	1.83						
**Level of functioning at baseline**								−0.10 (−0.30 to 0.11)			-0.02
	Low	25.02 (22.34 to 27.70)	1.37	22.27 (19.59 to 24.95)	1.37						
	Mean	24.07 (22.37 to 25.77)	0.87	22.65 (20.95 to 24.35)	0.87						
	High	23.12 (20.52 to 25.72)	1.33	23.03 (20.43 to 25.63)	1.33						
**Overall self-rated self-compassion at baseline**								0.06 (−0.83 to 0.94)			0.01
	Low	23.81 (19.66 to 27.95)	2.11	21.28 (17.14 to 25.43)	2.11						
	Mean	24.02 (22.29 to 25.76)	0.89	22.53 (20.79 to 24.26)	0.89						
	High	24.24 (20.74 to 27.74)	1.79	23.77 (20.27 to 27.28)	1.79						
**Momentary self-compassion at baseline**								−2.83 (−5.66 to 0.00)			-0.37
	Low	26.60 (23.53 to 29.68)	1.57	23.05 (19.97 to 26.12)	1.57						
	Mean	24.17 (22.47 to 25.88)	0.87	22.68 (20.98 to 24.38)	0.87						
	High	21.74 (18.88 to 24.60)	1.46	22.31 (19.45 to 25.17)	1.46						
**Adaptive emotion regulation at baseline**								−0.36 (−1.96 to 1.24)			−0.06
	Low	24.57 (21.68 to 27.45)	1.47	24.01 (21.13 to 26.90)	1.47						
	Mean	24.01 (22.35 to 25.74)	0.87	22.66 (20.96 to 24.63)	0.87						
	High	23.52 (20.64 to 26.41)	1.47	21.30 (18.42 to 24.19)	1.47						
**Maladaptive emotion regulation at baseline**								0.03 (−1.74 to 1.80)			0.00
	Low	24.00 (20.92 to 27.08)	1.57	20.53 (17.45 to 23.62)	1.57						
	Mean	24.05 (22.35 to 25.74)	0.87	22.66 (20.96 to 24.36)	0.87						
	High	24.09 (21.01 to 27.17)	1.57	24.78 (21.70 to 27.86)	1.57						

^a^Adjusted for baseline levels of psychological distress.

^b^d-type effect size.

^c^N/A: not applicable.

^d^Low = mean − 1 SD.

^e^High = mean + 1 SD.

^f^Stage 1a (individuals with psychological distress) is used as the reference category.

**Table 5:** Associations of general psychopathology with participants’ characteristics and level of putative mechanisms and processes at baseline and predicted marginal means^a^.

		Postintervention			Follow-up			Adjusted B (95% CI)			Effect size^b^	
		Predicted marginal mean (95% CI)	SE	Predicted marginal mean (95% CI)		SE						
Time		—^c^	—	—		—	−25.10 (−56.83 to 6.63)			−2.38		
**Age**									−0.79 (−2.05 to 0.46)			−0.08
	Low^d^	19.75 (15.20 to 24.29)	2.32	18.27 (13.72 to 22.81)		2.32						
	Mean	17.49 (14.58 to 20.39)	1.48	16.06 (13.15 to 18.96)		1.48						
	High^e^	15.23 (10.61 to 19.86)	2.36	13.85 (9.22 to 18.48)		2.36						
**Gender**									−2.14 (−10.25 to 5.97)			−0.20
	Female	18.01 (14.57 to 21.45)	1.76	17.44 (14.00 to 20.88)		1.76						
	Male	15.87 (8.96 to 22.78)	3.53	11.49 (4.58 to 8.40)		3.53						
**Ethnic minority status**									1.40 (−10.21 to 13.02)			0.13
	White majority	17.40 (14.30 to 20.49)	1.58	15.69 (12.60 to 18.78)		1.58						
	Minority	18.80 (7.84 to 29.75)	5.59	20.10 (9.15 to 31.06)		5.59						
**Clinical stage^f^**									−0.97 (−6.84 to 4.89)			−0.09
	Stage 1a	19.07 (15.13 to 23.01)	2.01	18.29 (14.35 to 22.23)		2.01						
	Stage 1b	14.32 (8.75 to 19.88)	2.84	11.60 (6.03 to 17.17)		2.84						
	Stage 2	17.82 (7.26 to 28.39)	5.39	16.34 (5.77 to 26.91)		5.39						
**Psychological distress at baseline**									−0.27 (1.55 to 1.01)			−0.03
	Low	18.83 (12.03 to 25.63)	3.47	19.44 (12.64 to 26.25)		3.47						
	Mean	17.52 (14.61 to 20.42)	1.48	16.07 (13.17 to 18.98)		1.48						
	High	16.20 (9.35 to 23.06)	3.50	12.71 (5.85 to 19.56)		3.50						
**Level of functioning at baseline**									−0.13 (−0.48 to 0.22)			−0.01
	Low	18.84 (14.26 to 23.43)	2.34	15.01 (10.43 to 19.60)		2.34						
	Mean	17.56 (14.65 to 20.46)	1.48	16.06 (13.16 to 18.97)		1.48						
	High	16.27 (11.82 to 20.72)	2.27	17.11 (12.66 to 21.57)		2.27						
**Overall self-rated self-compassion at baseline**									0.31 (−1.20 to 1.83)			0.03
	Low	16.18 (9.09 to 23.27)	3.62	10.90 (3.81 to 17.99)		3.62						
	Mean	17.39 (14.43 to 20.36)	1.51	15.60 (12.63 to 18.57)		1.51						
	High	18.61 (12.62 to 24.60)	3.05	20.29 (14.31 to 26.28)		3.05						
**Momentary self-compassion at baseline**									−3.24 (−8.08 to 1.61)			−0.31
	Low	20.45 (15.19 to 25.70)	2.68	15.26 (10.01 to 20.52)		2.68						
	Mean	17.67 (14.76 to 20.58)	1.49	16.05 (13.14 to 18.96)		1.49						
	High	14.89 (9.99 to 19.79)	2.50	16.84 (11.94 to 21.73)		2.50						
**Adaptive emotion regulation at baseline**									0.32 (−2.42 to 3.06)			0.03
	Low	17.06 (12.13 to 22.00)	2.52	19.47 (14.53 to 24.41)		2.52						
	Mean	17.52 (14.62 to 20.43)	1.48	16.09 (13.19 to 19.00)		1.48						
	High	17.98 (13.05 to 22.92)	2.52	12.71 (7.78 to 17.65)		2.52						
**Maladaptive emotion regulation at baseline**									0.97 (−2.42 to 3.06)			0.09
	Low	16.11 (10.84 to 21.38)	2.69	14.42 (9.15 to 19.69)		2.69						
	Mean	17.52 (14.62 to 20.43)	1.48	16.09 (13.19 to 19.00)		1.48						
	High	18.93 (13.67 to 24.20)	2.69	17.76 (12.49 to 23.03)		2.69						

^a^Adjusted for baseline levels of general psychopathology.

^b^d-type effect size.

^c^Not available.

^d^Low = mean – 1 SD.

^e^High = mean + 1 SD.

^f^Stage 1a (individuals with psychological distress) is used as the reference category.

## Discussion

### Principal Findings

First, we developed a hybrid 6-week CFI comprising 2 intervention tracks with varying foci and demand levels. Second, we observed initial signals of effects of age, general psychopathology, and clinical stage on change in momentary self-compassion and change in emotion regulation. Older participants tended to show greater differences in momentary self-compassion comparing baseline and postintervention assessments. Participants classified as stage 2 were found to show greater differences in adaptive emotion regulation comparing baseline and postintervention assessments. In addition, participants with lower levels of psychopathology at baseline showed more pronounced reductions in maladaptive emotion regulation from baseline to postintervention assessments. There was no evidence for associations of other baseline characteristics (eg, gender, minority status, and level of functioning) and putative mechanisms (ie, overall self-rated self-compassion, working alliance, and training frequency). Third, there was some evidence that higher momentary self-compassion at baseline tended to be associated with, on average, lower levels of psychological distress across postintervention and follow-up assessments. We observed no other initial signals that clinical or functional characteristics at baseline impacted clinical outcomes.

### Methodological Considerations

The reported results should be interpreted in light of several methodological considerations and limitations. First, sample size and selection as well as the exploratory nature of the analyses need to be critically appraised. Although the analyses were prospectively registered, they reflect secondary analyses with an increased risk of type 1 error. As noted, our findings reflect initial signals of associations of participants’ baseline characteristics with putative mechanisms, processes, and outcomes. Moreover, it should be taken into account that boys or men, individuals identifying as nonbinary, and participants from stage 2 (first episode of severe mental disorder) were considerably underrepresented in the sample. However, the gender difference in recruitment may partly be explained by higher prevalence of depressive and anxiety disorders in women and adolescent girls [83,84] and the exclusion of mental health problems that are especially prevalent in men and adolescent boys (eg, primary substance abuse disorder [66]). Randomization in a future definitive trial may therefore need to stratify by gender to rule out potential confounding by this factor. In addition, we assessed ethnicity by considering participants’ self-report of citizenship, country of birth, first language, and information provided in participants’ family assessment. Grouping participants into broad categories of ethnicity inevitably implies that some participants may have been assigned to a category that they do not consider belonging to and, hence, misclassification. In general, the concept of using categories, for example, with regard to ethnicity or gender, may be criticized as—of course—there is considerable heterogeneity within groups, which needs to be further explored in qualitative analyses [85,86]. These limitations can be tolerated at the exploratory stage of developing a complex intervention but should be addressed in future, definitive trials.

Second, operationalizations of putative mechanisms were not measured at multiple time points during the intervention, and difference scores were used as proxies for change in self-compassion and emotion regulation. While proxies are acceptable in this exploratory study, a future definitive trial may use multiple assessments during the intervention to yield more fine-grained data on potential changes in mechanisms.

Third, the assessment of self-compassion needs to be critically appraised: in our analyses, overall self-rated self-compassion and momentary self-compassion were not correlated, indexing low convergent validity (Multimedia Appendix 5). Similar phenomena have been observed before, for example, for negative symptoms measured with EMA and interviewer-rated measures, which may tap distinct but related constructs [87]. This may be viewed as underscoring the relevance of assessment under real-time and real-world conditions, which is supported by moderate to large correlations of momentary self-compassion with clinical characteristics (ie, clinical stage, psychological distress, general psychopathology, and level of functioning), indicating high concurrent validity. However, as the items for assessing momentary self-compassion were used for the first time in this study, they may also not fully capture the construct of self-compassion as operationalized by the subscales in the Self-Compassion Scale (ie, they are more similar in content to items from the self-kindness than mindfulness subscale) [73]. In addition, we aggregated EMA data on momentary self-compassion at the person level, which led to a loss of information in comparison with the level of EMA observations, given the repeated measurement and temporal variability EMA captures as an intensive longitudinal data collection method (Schick A, unpublished data, 2022). Nonetheless, aggregated experience sampling measures may still capture the target constructs with less noise and greater sensitivity than recall measures [88], so this may not reduce this study’s informative value substantially.

Fourth, potential influences of the COVID-19 pandemic have not been statistically accounted for in current analyses and should be considered when interpreting the findings. Owing to local regulations (eg, lockdowns and contact restrictions), the intervention sessions were shifted from face-to-face contact to video calls. Recent systematic reviews and meta-analyses indicated no differences in telehealth and in-person psychotherapy [89], but generalizability to settings in which both in-person sessions and a video call format are used flexibly remains unclear, and the impact cannot be determined with certainty without further research.

### Comparison With Previous Research

To our knowledge, the EMIcompass intervention is the first hybrid CFI blending an EMI and face-to-face sessions designed to enhance self-compassion and resilience in young people with nonspecific psychological distress, CHARMS, and first episode of severe mental disorder. Building on principles of EMIs [26,27,29,34], EMIcompass combined different intervention elements: enhancing tasks provided participants with new CFI strategies. Consolidating tasks facilitated training in different contexts and translation into daily life increasing the chances of generalization. Elements of experience sampling were used to increase reflective processing improving insight and awareness of own cognitive and emotional processes [90]. This may be further improved by incorporating elements of feedback into future versions of the intervention [91]. In addition, assessing stress and affect in daily life allows the EMI to offer useful techniques in moments of high distress (ie, interactive tasks), providing participants with support in challenging life situations.

For the EMIcompass intervention, the results from an uncontrolled pilot study [54] indicated a reduction of stress reactivity at postintervention and follow-up and reduced clinical symptoms at follow-up when compared with baseline. A recent exploratory RCT [56] indicated that all feasibility criteria were met and a reduction of stress reactivity in the experimental condition as the primary candidate mechanism in comparison with a control condition of TAU. In addition, it suggests initial signals that the EMIcompass intervention may have beneficial effects on resilience in daily life and quality of life. Detailed findings on feasibility and initial signals of efficacy are described elsewhere [56].

Apart from an association of age and change in momentary self-compassion, participants’ sociodemographic characteristics were not associated with putative processes, mechanisms, and outcomes of the EMIcompass intervention. This is at variance with findings in traditional psychotherapy for depression and psychosis, where reviews indicate differential treatment effects for various sociodemographic characteristics (eg, age, gender, and marital status) [92,93]. In an Acceptance and Commitment Therapy–based EMI in individuals at ultra–high risk for psychosis and with a first episode of psychosis, ethnic minority status was associated with lower compliance and higher app usefulness, whereas being female predicted lower usefulness of the app’s metaphor images (van Aubel E, unpublished data, August 2022).

When examining the impact of clinical and functional characteristics, we observed associations of clinical stage and general psychopathology with putative mechanisms and processes (ie, change in momentary self-compassion and change in emotion regulation). Interestingly, *later* clinical stage was associated with a more pronounced increase in adaptive emotion regulation, whereas *lower* levels of general psychopathology tended to be associated with a more pronounced reduction of maladaptive emotion regulation. However, the findings on clinical stage must be interpreted with caution, given the small number of participants from stage 2 included in the study. The possibility of ceiling effects for a particular clinical stage could be ruled out, as the mean levels of adaptive emotion regulation were in the middle range of the scale for all clinical stages. An RCT of cognitive behavioral therapy in patients with psychotic disorders investigating predictors of improvement and dropout indicated that higher symptom severity and poor level of functioning do not pose a barrier to improvement [94]. The findings from an Acceptance and Commitment Therapy–based EMI in individuals at ultra–high risk for psychosis and with a first episode of psychosis show a differentiated perspective on symptom severity: the severity of affective symptoms was associated with higher perceived usefulness and that of negative symptoms was associated with lower perceived usefulness of the intervention (van Aubel E, unpublished data, August 2022). Besides sociodemographic, clinical, and functional characteristics at baseline, we moved beyond these previous studies and examined potential associations of baseline levels of self-compassion and emotion regulation with outcomes of the intervention. We found some evidence that higher levels of momentary self-compassion at baseline were associated with, on average, lower levels of psychological distress across assessment time points. By showing this in a longitudinal intervention study, the current findings extend evidence from a meta-analysis indicating associations of self-compassion and psychological distress in general [95]. However, in this study, this did not hold true for overall self-compassion. Apart from the effects delineated earlier, there were no initial signals of associations, tentatively suggesting that participants’ sociodemographic, clinical, and functional characteristics had little influence on their response to the EMIcompass intervention. This may indicate—within the limits of the variables assessed—that the EMIcompass intervention is relatively inclusive and reach of participants is largely independent from their sociodemographic, clinical, and functional baseline characteristics.

The role of digital approaches in improving the reach of those in need within broader conceptualizations has been subject to controversial debate: qualitative studies with health professionals and service users indicate that digital approaches were viewed as having the potential to improve inclusion but also as having the risk of digital exclusion [36,37,96]. Concerns have been raised that digital approaches and the digital divide may further reinforce health inequalities (ie, systematic, avoidable, and unfair differences in health outcomes [97]) in marginalized and underserved populations; for example, in racial and ethnic minorities [98]. Digital inequalities are suggested to comprise multiple continuous dimensions; for example, socioeconomic and educational background, migrant and ethnic minority status, and health literacy [99-101]. To further improve our understanding of the consequences of digital inequalities for individuals’ response to the EMIcompass intervention, future studies may broaden their perspective by including further aspects of marginalized and underserved populations (eg, sexual minority status and socioeconomic background) and examining other criteria (eg, level of functioning, satisfaction with the intervention, goal attainment, and quality of life) in addition to those considered so far.

To address digital exclusion of marginalized and underserved populations, demands for evidence-based digital inclusion strategies have been articulated [102], and potential pathways for improving inclusion in digital approaches have been discussed. On the one hand, adaptations of interventions have been suggested; for example, feasibility and beneficial effects of cultural adaptation of interventions have already been demonstrated [103]. In addition to adapting interventions for specific groups, the needs and perspectives of individual participants should be taken into account in process evaluations combining quantitative and qualitative data [104]. In line with this, we conducted a qualitative study incorporating realist methodology [105] examining what works for whom under which circumstances in the EMIcompass study, the findings of which are reported elsewhere (Paetzold I, unpublished data, 2022). An emerging research field targets the adaptation of digital interventions on an individual level aiming at personalizing assessment and intervention [27,29,34]. On the other hand, the creation of interventions for diverse populations has been suggested, for example, in the REACT recommendations [98]. In line with this approach, a recent review of digital mental health interventions specifically designed for marginalized populations indicated promising results on feasibility and acceptability in pilot studies but also a lack of larger-scale examinations [106].

### Conclusions

We developed the first hybrid CFI combining an EMI and face-to-face sessions with 2 intervention tracks and varying foci and demand levels to enhance resilience in young people with early mental health problems. We aimed at exploring whether participants’ characteristics at baseline were associated with putative mechanisms and outcomes of the EMIcompass intervention. The findings indicated reach of participants by the intervention largely independent of sociodemographic, clinical, and functional baseline characteristics. The findings need to be confirmed in a definitive trial.

## Appendix

Multimedia Appendix 1 Changes to the pilot version.

Multimedia Appendix 2 EMIcompass intervention manual.

Multimedia Appendix 3 Modified criteria of the clinical staging model.

Multimedia Appendix 4 Measures.

Multimedia Appendix 5 Correlation table.

Multimedia Appendix 6 Variance inflation factors and tolerance.

Multimedia Appendix 7 Cross-differences.

## Abbreviations

CFI: compassion-focused intervention
CHARMS: Clinical High At-Risk Mental State
EMA: ecological momentary assessment
EMI: ecological momentary intervention
ESM: experience sampling methodology
HiTOP: Hierarchical Taxonomy of Psychopathology
mHealth: mobile health
RCT: randomized controlled trial
TAU: treatment as usual
