# Supplementary material for: A Hybrid Ecological Momentary Compassion–Focused Intervention for Enhancing Resilience in Help-Seeking Young People: Prospective Study of Baseline Characteristics in the EMIcompass Trial
Source: JMIR Form Res. 2022 Nov 4;6(11):e39511. doi: 10.2196/39511 (PMC9675017; doi:10.2196/39511)
Supplement: Multimedia Appendix 1 [file formative_v6i11e39511_app1.docx]

Multimedia Appendix 1 – Measures

Table S1. Description of measures and time points of their administration.

| Measures | Assessment method | Instrument und further information ^a^ | Time point |
| --- | --- | --- | --- |
| **Sociodemographic characteristics** | | | |
| *Age* | Self-report |  | Screening |
| *Gender* | Self-report |  | Screening |
| *Ethnicity* | Self-report, family assessment | Citizenship, country of birth, first language and family assessment. | Screening, T0 |
| ***Clinical characteristics*** | | | |
| *Clinical stage* | Standardized interviews,  self-report, observer ratings | Allocation to stage 1a (i.e., current psychological distress), stage 1b (i.e., broad Clinical High At-Risk Mental State (CHARMS) with attenuated symptoms of psychosis, mania, depression, or anxiety) or stage 2 (i.e., first episode of severe mental disorder) based on a modified version of the clinical staging model [1, 2].  Further details on the criteria are provided elsewhere [1] and in Multimedia Appendix 4. | Screening |
| *Psychological distress* | Self-report questionnaire | Assessment with the Kessler Psychological Distress Scale (K10) [3, 4], a 10-item questionnaire assessing psychological distress in the last month on a scale from 1 (= never) to 5 (= always).  To obtain a measure of psychological distress, a sum score across all items was calculated. α = .73 at baseline. | Screening, T1, T2 |
| *General psychopathology* | Self-report questionnaire | Assessment with the 18-item version of the Brief Symptom Inventory (BSI-18) [5, 6]. Participants were asked to rate to what extend they experienced the listed 18 symptoms in the last seven days on a 5-point Likert-scale ranging from 0 (= not at all) to 4 (= very strong).  We used the Global Severity Index, the sum score across all items, as a measure of general psychopathology. α = .79 at baseline. | T0, T1, T2 |
| *Level of functioning* | Observer rating | Trained assessors rated participants’ level of functioning using the Social and Occupational Functioning Assessment Scale (SOFAS) [7]. Ratings range from 0 to 100, with lower scores indicating lower levels of functioning.  A random set of audiotaped interviews rated by a fixed set of trained raters was used to determine interrater-reliability using JASP (i.e., ICC3.1, JASP version 0.16.1 [8, 9]). In line with the literature [10], we observed excellent interrater-reliability (ICC 0.96; 95%CI 0.86-0.99). | Screening |
| **Baseline levels of putative mechanisms** | | | |
| *Self-compassion* |  | Overall self-rating and momentary EMA ratings in participants’ daily life.  To indicate change in self-compassion from baseline to post-intervention, we calculated difference scores (δ_post-intervention – baseline_). |  |
| *Overall self-compassion* | Self-report questionnaire | Assessment with the Self-Compassion Scale [11] with 26 items. In line with Neff [12], we re-coded items from the subscales self-judgement, isolation and over-identification. We then calculated a mean for each of the subscales, and finally summed the means to create a total self-compassion score.  α = .90 at baseline. | T0, T1 |
| *Momentary self-compassion* | EMA | Three items: “I feel safe.”, “I feel benevolent.”, “I like myself.”, 7-point Likert-scale ranging from 1 (= not at all) to 7 (= very much). Assessments eight times a day at six consecutive days at baseline and post-intervention.  We aggregated participants’ ratings of momentary self-compassion to a mean score for each assessment week (baseline, post-intervention).  α = .84 at baseline. | T0, T1 |
| *Emotion regulation* | Self-report questionnaire | Assessment with the Cognitive Emotion Regulation Questionnaire (CERQ-short), an 18-item questionnaire capturing nine strategies (self-blame, other-blame, rumination, catastrophizing, positive refocusing, planning, positive reappraisal, putting into perspective and acceptance) on a 5-point Likert scale ranging from 1 (= almost never) to 5 (= almost always) [13]. In line with the CERQ scoring manual [14], we calculated sum scores for each subscale. Following the approach of Martins, Freire [15], we classified the coping strategies as adaptive (acceptance, putting into perspective, positive refocusing, refocus on planning and positive reappraisal) or maladaptive (self-blame, rumination, catastrophizing and other-blame). We aggregated the values to a mean score for adaptive and a mean score for maladaptive coping strategies. Adaptive emotion regulation α = .82, maladaptive emotion regulation α = .77 at baseline.  To indicate change in emotion regulation from baseline to post-intervention, we calculated a difference score (δ_post-intervention – baseline)._ | T0, T1 |
| *Working alliance* | Self-report questionnaire | Assessment with the Working Alliance Inventory for Patients and Therapists (WAI-P / WAI-T, [16]). We used sum scores of the 12 items for patient and therapist ratings to obtain measures of working alliance. Patient ratings: α = .92, therapist ratings α = .93. | T1 |
| *Training frequency* |  | Total number of training tasks completed in the EMI. | Intervention period |

### ^a^ A correlation table of the measures used is displayed in Multimedia Appendix 5.

^b^ T0 = baseline assessment, T1 = post-intervention assessment, T2 = follow-up assessment.

1. Schick A, Paetzold I, Rauschenberg C, Hirjak D, Banaschewski T, Meyer-Lindenberg A, et al. Effects of a Novel, Transdiagnostic, Hybrid Ecological Momentary Intervention for Improving Resilience in Youth (EMIcompass): Protocol for an Exploratory Randomized Controlled Trial. JMIR Res Protoc. 2021 Dec 3;10(12):e27462. PMID: 34870613. doi: <https://doi.org/10.2196/27462>.

2. Hartmann JA, Nelson B, Spooner R, Paul Amminger G, Chanen A, Davey CG, et al. Broad clinical high-risk mental state (CHARMS): Methodology of a cohort study validating criteria for pluripotent risk. Early Interv Psychiatry. 2019 Jun;13(3):379-86. PMID: 28984077. doi: <https://doi.org/10.1111/eip.12483>.

3. Kessler RC, Andrews G, Colpe LJ, Hiripi E, Mroczek DK, Normand S-L, et al. Short screening scales to monitor population prevalences and trends in non-specific psychological distress. Psychol Med. 2002;32(6):959-76.

4. Kessler RC, Berglund P, Demler O, Jin R, Merikangas KR, Walters EE. Lifetime prevalence and age-of-onset distributions of DSM-IV disorders in the National Comorbidity Survey Replication. Arch Gen Psychiatry. 2005;62(6):593-602. doi: <https://doi.org/10.1001/archpsyc.62.6.593>.

5. Derogatis LR. BSI brief symptom inventory. Administration, scoring, and procedures manual. Minneapolis, MN: National Computer Systems.; 1993.

6. Derogatis LR, Fitzpatrick M. The SCL-90-R, the brief symptom inventory (BSI), and the BSI-18. In: Maruish ME, editor. The use of psychological testing for treatment planning and outcomes assessment: Instruments for adults Mahwah, NJ: Lawrence Erlbaum Associates Publishers; 2004. p. 1–41.

7. Goldman HH, Skodol AE, Lave TR. Revising axis V for DSM-IV: A review of measures of social functioning. Am J Psychiatry. 1992;149:9. doi: 10.1176/ajp.149.9.1148.

8. Shrout PE, Fleiss JL. Intraclass correlations: Uses in assessing rater reliability. Psychol Bull. 1979;86(2): 420–8. doi: <https://doi.org/10.1037/0033-2909.86.2.420>.

9. JASP Team. JASP (Version 0.16.1). 2022.

10. Hilsenroth MJ, Ackerman SJ, Blagys MD, Baumann BD, Baity MR, Smith SR, et al. Reliability and Validity of DSM-IV Axis V. Am J Psychiatry. 2000;157(11):1858-63. PMID: 11058486. doi: 10.1176/appi.ajp.157.11.1858.

11. Hupfeld J, Ruffieux N. Validierung einer deutschen version der Self-Compassion Scale (SCS-D). Z klin Psychol Psychother. 2011. doi: 10.1026/1616-3443/a000088.

12. Neff KD. The development and validation of a scale to measure self-compassion. Self Identity. 2003;2(3):223-50. doi: 10.1080/15298860309027.

13. Garnefski N, Kraaij V. Cognitive emotion regulation questionnaire–development of a short 18-item version (CERQ-short). Pers Individ Differ. 2006;41(6):1045-53. doi: 10.1016/j.paid.2006.04.010.

14. Garnefski N, Kraaij V, Spinhoven P. CERQ - Manual for the use of the Cognitive Emotion Regulation Questionnaire. Leiderdorp, The Netherlands: DATEC; 2002.

15. Martins E, Freire M, Ferreira-Santos F. Examination of adaptive and maladaptive cognitive emotion regulation strategies as transdiagnostic processes: associations with diverse psychological symptoms in college students. Stud Psychol. 2016;58(1).

16. Horvath AO, Greenberg LS. Development and validation of the Working Alliance Inventory. J Couns Psychol. 1989;36(2):223.
