# Supplementary material for: A Hybrid Ecological Momentary Compassion–Focused Intervention for Enhancing Resilience in Help-Seeking Young People: Prospective Study of Baseline Characteristics in the EMIcompass Trial
Source: JMIR Form Res. 2022 Nov 4;6(11):e39511. doi: 10.2196/39511 (PMC9675017; doi:10.2196/39511)
Supplement: Multimedia Appendix 2 [file formative_v6i11e39511_app2.docx]

# Multimedia Appendix 2 – Changes to the pilot version

- Extended intervention period from 3 to 6 weeks, more strategies/tasks presented
- Two intervention tracks with varying foci and demand levels
- Sessions delivered in face-to-face contact or via video calls
- To lower the burden: reduced number of interactive questionnaires, morning and evening questionnaire omitted
- Gamification elements
- Moved from Psymate to movisensXS
- Translation for Dutch to German
