# Supplementary material for: A Hybrid Ecological Momentary Compassion–Focused Intervention for Enhancing Resilience in Help-Seeking Young People: Prospective Study of Baseline Characteristics in the EMIcompass Trial
Source: JMIR Form Res. 2022 Nov 4;6(11):e39511. doi: 10.2196/39511 (PMC9675017; doi:10.2196/39511)
Supplement: Multimedia Appendix 3 [file formative_v6i11e39511_app3.docx]

## Multimedia Appendix 3 – EMIcompass intervention manual

## **Introduction**

EMIcompass is an outpatient intervention that targets adolescents and young adults with multiple mental health difficulties. The target group includes adolescents and young adults seeking help with nonspecific symptoms, individuals at increased risk of developing a mental disorder, and patients with a first-time mental illness. EMIcompass is a guided, application-based (app), mHealth self-help intervention that lasts a total of six weeks and includes three therapeutic sessions and a closing session. The app transmits and visualizes therapeutic content during and between therapy sessions. In addition, daily exercises to reinforce what has been learned are offered via the app between sessions. Optionally, there is also the possibility to record the mental state of the participants in everyday life, in order to offer helpful exercises, if necessary.

EMIcompass is based on the rationale of Compassion Focused Therapy (CFT; Gilbert, 2009, 2013) and aims to promote a compassion-based, benevolent self-image and to strengthen the capacity for self-care in everyday life. CFT techniques are utilized, such as various breathing and imagination exercises.

## Core principles of Compassion Focused Therapy (Gilbert, 2009, 2013)

Gilbert (2013), drawing on a quote from the Dalai Lama, refers to compassion as "sensitivity to one's own suffering and that of others, with a deep dedication to alleviating it."

CFT aims to foster this compassion for the self and others. Self-compassion is referred to in the intervention as "self-care" or "compassionate or benevolent treatment of oneself" to make the terminology simpler and more catchy for adolescents (Gilbert, 2013).

Findings from evolutionary biology show that various psychological phenomena arose at different times in evolution. Competencies such as sexuality, fighting, defending oneself and one's territory, etc. emerged early.

Humans share these basal survival skills with many other creatures. Abilities such as complex thinking and reasoning, reflection, theory of mind, and sense of self-identity emerged much later. Consequently, the human brain combines a variety of different motives and emotions, which can conflict with each other. Thus, according to Gilbert (2013), many psychological problems can be seen as unhelpful, conflicting circuits between old and newer systems.

Physical symptoms, for example, in conjunction with the newer skills of thinking, reasoning, and explaining, can lead to the conclusion "my racing heart means I'm going to have a heart attack and die," which in turn can trigger a panic attack. Gilbert (2013) thus describes the human brain as a "*tricky brain*."

CFT (Gilbert, 2009, 2013) assumes three basic emotion systems in which emotions can be grouped according to their function. The three emotion systems are:

- *threat* (danger): the threat emotion system includes emotions such as anger, fear, and disgust, functions that help us recognize danger and protect ourselves.

- *drive* (goals and needs): The goals and needs emotion system includes emotions such as excitement and joy, functions that motivate us to move toward helpful goals and resources.

- soothing (calm and security): The calm and security emotion system includes emotions such as satisfaction and security, functions that motivate us to take care of ourselves and others and to allow others to take care of us.

Many participants have a very active danger system and are therefore often and strongly confronted with emotions such as anger and fear. The therapeutic elements of CFT (Gilbert, 2009, 2013) strengthen the calm and security system to mitigate negative emotions from the danger system and form a basis for emotions from the goal and need system.

## The basic therapeutic attitude of EMIcompass

The basic therapeutic attitude of the EMIcompass sessions is also based on the therapeutic rationale of CFT (Gilbert, 2009, 2013). Thus, a warm, unconditionally appreciative, and empathic attitude of the therapist is of utmost relevance. This is expressed in the following principles according to which sessions should be designed:

- Take time to listen to participants' concerns and problems, make them feel that you are listening with genuine interest
- Address all participants' reactions compassionately ("leading by example")
- A focus of the intervention is "de-shaming," so validate participants' individual emotional experiences sincerely
- Understand safety strategies and symptoms as the participant's best effort to deal with difficult situations
- Be careful to show compassion but not pity
- Be patient and do not try to force anything
- Exude warmth and friendliness
- Anticipate and validate participant resistance and acknowledge that it can be very difficult for participants
- Make participants feel that there is no pressure. Convey the feeling that it is perfectly okay, and you understand if they have difficulty doing the exercises

## Instructions for the use of the manual for study therapists

All sessions in the EMIcompass intervention are described and explained in this manual. The manual contains sample phrases that you can use in your sessions.

*Only relevant if intervention is held in German:*

In the sample formulations, participants are addressed by their first name, but for adolescents under 18 years of age, "Du" may of course be the more appropriate form of address; with adolescents and young adults who are currently in transition, the form of address can also be briefly agreed upon at the beginning of the sessions.

The manual is structured to provide a brief overview at the beginning of each session, summarizing the planned structure and time approaches. The individual sessions are divided into subsections. In addition to the introduction and conclusion of the sessions, there will be phases of information transfer - hereafter referred to as **info** and marked with this symbol [
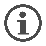
](https://www.google.com/url?sa=i&rct=j&q=&esrc=s&source=images&cd=&cad=rja&uact=8&ved=2ahUKEwj6iKDkjYLhAhUIDuwKHYniA0oQjRx6BAgBEAU&url=https://de.wikipedia.org/wiki/Datei:Infobox_info_icon.svg&psig=AOvVaw379mimxNArReUBgp9p_TUm&ust=1552669234895208) .

In addition, the intervention includes concrete **exercises** that are carried out together in the sessions, marked below with this symbol [
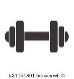
](https://www.google.com/url?sa=i&rct=j&q=&esrc=s&source=images&cd=&cad=rja&uact=8&ved=2ahUKEwiZj8uHjoLhAhXOy6QKHX8wAOYQjRx6BAgBEAU&url=https://www.fotosearch.de/clip-art/hantel.html&psig=AOvVaw1I-dWoT1U0d0S0a77ermuV&ust=1552669309693258). During the sessions, you first discuss the small information section with the participants and then jointly perform the associated exercise on the smartphone. It is important that the participants work through the exercises on the smartphone themselves, while you guide them in the therapeutic conversation. You are the contact person for any uncertainties and questions.

EMIcompass aims to provide optimal support for all participants, which is why the intervention is divided into two levels. Level 1 contains somewhat easier exercises and is aimed at participants who find imagination exercises difficult (the exercises are marked with X.1). The more demanding Level 2 is aimed at participants who find imagination exercises easy (the exercises are marked with X.2). The Assignment to the study arms will be made in the second interview session and will be based on the participants' report and your clinical impression.

For participants to progress to the next week of intervention and learn a new exercise, it is necessary that the introduction of the new exercise has been made and practiced at least once during the week. If this is not the case, participants remain in the week and repeat it, which is set by the study therapist (see technique guide for instructions).

# EMIcompass Intervention – Overview

| **Week** | **Level 1** | | **Level 2** |
| --- | --- | --- | --- |
| 1 | 1. Emotional compass  2. Count the breath | | |
| 2 | 3. Find a soothing color | | |
| 3 | 4.1 Find a calm and safe place | 4.2 Compassionate companion | |
| 4 | 5.1 Breathing with pauses | 5.2 Find a calm and safe place | |
| 5 | 6.1 Surf the waves of your feelings | 6.2 My compassionate self | |
| 6 | 7.1 My toolbox | 7.2 My compassionate message | |
|  | Closing session after week 6 | | |

# EMIcompass intervention - implementation of the sessions

## First session (1. week)

### Overview of the first session

|  |  |
| --- | --- |
| Introduction | 5 min |
| Getting to know, overview of complaints | 10 min |
| [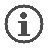](https://www.google.com/url?sa=i&rct=j&q=&esrc=s&source=images&cd=&cad=rja&uact=8&ved=2ahUKEwj6iKDkjYLhAhUIDuwKHYniA0oQjRx6BAgBEAU&url=https://de.wikipedia.org/wiki/Datei:Infobox_info_icon.svg&psig=AOvVaw379mimxNArReUBgp9p_TUm&ust=1552669234895208) Information: Emotional compass | 10 min |
| [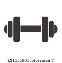](https://www.google.com/url?sa=i&rct=j&q=&esrc=s&source=images&cd=&cad=rja&uact=8&ved=2ahUKEwiZj8uHjoLhAhXOy6QKHX8wAOYQjRx6BAgBEAU&url=https://www.fotosearch.de/clip-art/hantel.html&psig=AOvVaw1I-dWoT1U0d0S0a77ermuV&ust=1552669309693258)Exercise: Emotional compass | 10 min |
| [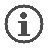](https://www.google.com/url?sa=i&rct=j&q=&esrc=s&source=images&cd=&cad=rja&uact=8&ved=2ahUKEwj6iKDkjYLhAhUIDuwKHYniA0oQjRx6BAgBEAU&url=https://de.wikipedia.org/wiki/Datei:Infobox_info_icon.svg&psig=AOvVaw379mimxNArReUBgp9p_TUm&ust=1552669234895208) Information: Count the breath | 5 min |
| [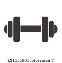](https://www.google.com/url?sa=i&rct=j&q=&esrc=s&source=images&cd=&cad=rja&uact=8&ved=2ahUKEwiZj8uHjoLhAhXOy6QKHX8wAOYQjRx6BAgBEAU&url=https://www.fotosearch.de/clip-art/hantel.html&psig=AOvVaw1I-dWoT1U0d0S0a77ermuV&ust=1552669309693258) Exercise: Count the breath | 5 min |
| Closing of the session | 5 min |

### Introduction

- Greeting, briefly introducing yourself
  - “*My name is XXX, I am a psychologist. Nice to meet you!"*
- Give a brief overview of the process
  - “*I would like to use the session today to get to know you and to discuss with you exactly what EMIcompass involves and how the treatment will proceed within the framework of EMIcompass. In addition, we can already start with some exercises today."*
- Study information:
  - *"As you heard in the preliminary interview, in this smartphone-assisted treatment I would like to offer to work with you over the next 6 weeks on improving your self-care in dealing with feelings in everyday life. Or perhaps to put it another way, I would like to offer that we practice together how to look at yourself a little more benevolently - just as a friend might. This will help you go through everyday life with a better emotional compass - which is why we call the treatment "EMICOMPASS". Through specific exercises, we will work on strengthening your self-care, sense of self-acceptance, and positive feelings in general. By self-care, I mean the ability to empathize with, understand and accept your own situation from your unique life story - so you learn to be more compassionate with yourself. For this, firstly, we will meet here every 2 weeks for a face-to-face session. I will also provide you with an app that can help you develop your emotional compass. The app will use signals to prompt you to do exercises and answer short mood queries, like the ones you already know from the last few days. If you want, you can also do more exercises on your own at any time or repeat exercises you have already done, you don't have to wait specifically for the app to prompt you through a signal. So, you can work on improving your self-care in dealing with feelings at any time* (use DEMO expression to explain the different categories of exercises). *However, please do not turn off your smartphone and charge it regularly. In the time between our conversations, I will support you by calling or emailing you to do the exercises on your own using the app. Do you agree with this? Do you have any questions?"*

### Getting to know, overview of complaints

- Getting to know each other and building relationships
  - *“Before we start with the exercises, I'd like to get to know you a bit first. Perhaps you would like to just briefly introduce yourself?"*
- Provide overview of life situation: student/apprentice/working? Leisure activities/hobbies? Who are important people in life, friends, family, partnership, etc.? Ask interested follow-up questions, active listening!
  - *“Thank you for telling me a little bit about yourself, I look forward to working with you over the next few weeks."*
- Overview of the complaints
  - *“How did it come about that you contacted us here at ZI? What was the reason?"* 🡪 Record reason for call and complaints so they can be used as examples later in the process. Validate remarks.
  - *"It would be important from our side that you please do not use drugs during the study, as they have a negative impact on your well-being and mental health."*

### *Emotional compass*

#### [
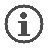
](https://www.google.com/url?sa=i&rct=j&q=&esrc=s&source=images&cd=&cad=rja&uact=8&ved=2ahUKEwj6iKDkjYLhAhUIDuwKHYniA0oQjRx6BAgBEAU&url=https://de.wikipedia.org/wiki/Datei:Infobox_info_icon.svg&psig=AOvVaw379mimxNArReUBgp9p_TUm&ust=1552669234895208)Information: Emotional compass

Exercise Introduction:

- *“Do you have any questions so far?* (If yes, answer questions, If no continue*). Then let's start with the exercises now!"*
- *“For this we will first familiarize ourselves with the app!* (Participant uses the smartphone him/herself, study therapist explains 🡪 DEMO printout can also be used)*. When you open the app, you will immediately see the home screen. New exercises always appear here on the home screen of the app. Look, here is the first exercise we will do together. It's called "New: Emotional compass. Please click on the exercise!"* Once the exercise is open, on the first page, briefly explain the structure of the exercises in the app*: "Here you can see the home screen of the first exercise, it is called "Emotional compass". At the top right you will see a small arrow, you can use it to scroll to the next page. Sometimes you can scroll down the pages, you'll see that later."*

Getting started with providing information, clicking through the app together with the patient while discussing the content:

- *"The exercise is about understanding our emotions better. That's why it's worth taking a brief look at our brain. As you know, humans have evolved over many thousands of years. From our ancestors, we have inherited an ancient brain, which is how other animals, even crocodiles, have it."* (click to continue)
- *“What can our old brain do now? The old brain has many important tasks, it is responsible for our emotions, such as fear, anger, or joy. It is also important for our behavior, for example whether we flee when it becomes dangerous. In addition, the old brain is responsible for our basic needs, for example eating or avoiding injury. These are all abilities that not only humans have, but also crocodiles."* (click to continue)
- *"Unlike the crocodile, we also have a new brain in addition, which developed much later in evolution. This distinguishes us from other animals. So, what can our new brain do? The new brain is responsible for our complex thinking, planning, and thinking about our thinking. It is through these abilities that the many great inventions that humans have made have come into being. However, worries and self-criticism also arise in the new brain. The crocodile can't build rockets to fly into space and it didn't invent the Internet, but it probably doesn't worry about the future either and probably doesn't suffer from self-doubt."* (click to continue)
- *“Between our old brain and our new brain, there are helpful circuits. But there are also unhelpful circuits. We call these circuits* ***'tricky brain loops.'*** *We just have a complicated brain."* (click to continue)
- *“Look at an example of an unhelpful cycle: Julia sees a photo on Facebook of several good friends having fun at another friend's party. The comment on the photo reads, 'All best friends together - what a party!!!' Upon seeing the photo, Julia begins to feel tense and anxious. A cycle develops between Julia's old brain and her new brain'* 🡪 Click through and discuss cycle
- *“Now you may wonder why we have these unhelpful circuits between the old brain and the new brain in the first place. The old brain wants to protect us from danger. That doesn't always fit well with what's going on in our new brain, which is what we think about situations in everyday life, for example. This creates unhelpful circuits.“*
- *“Perhaps you also know such or similar cycles from yourself? It's not your fault at all if you sometimes have similar thoughts and feelings as Julia in the example. We just have a tricky brain, a complicated brain."* (click to continue)
- *“The emotions that arise in our "tricky brain" can be thought of as a compass. Our* ***emotional compass*** *consists of* ***three emotion areas: Danger, Calm and Security****, and* ***Goals and Needs****, which influence each other. Let's take a closer look at these emotion areas together!"*
-
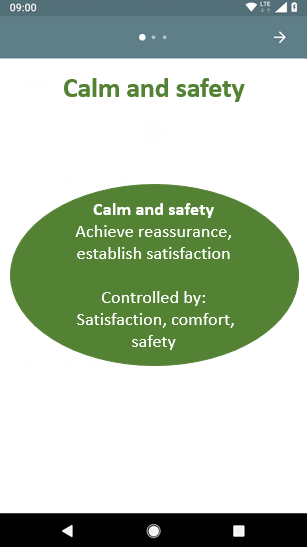

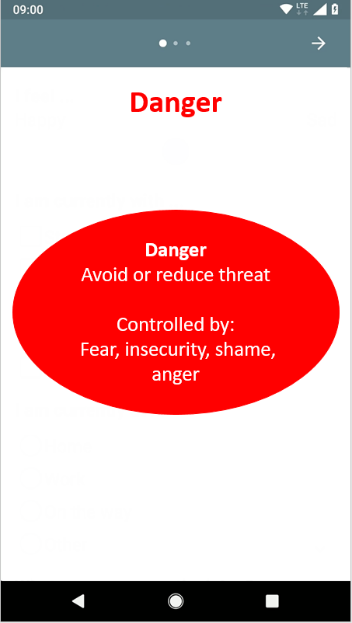

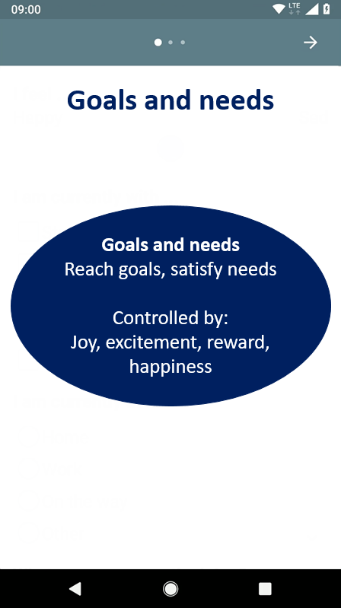
Introducting emotion areas:

After that, *"Do you have any questions about the emotion areas so far?"* (Answer questions, then click on next)

- *“Our emotion areas are interrelated and influence each other."* (click on next)
- *“Often our emotional compass is not very balanced. Red is often strongest and dominates our other emotion areas. Perhaps you know this about yourself?"* (click to continue)
- *“The red emotion area influences our thinking and behavior. It affects our attention, motivation and imagination. Red can often determine how our entire emotional compass works!"*

#### [
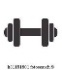
](https://www.google.com/url?sa=i&rct=j&q=&esrc=s&source=images&cd=&cad=rja&uact=8&ved=2ahUKEwiZj8uHjoLhAhXOy6QKHX8wAOYQjRx6BAgBEAU&url=https://www.fotosearch.de/clip-art/hantel.html&psig=AOvVaw1I-dWoT1U0d0S0a77ermuV&ust=1552669309693258) Exercise: Emotional compass

-
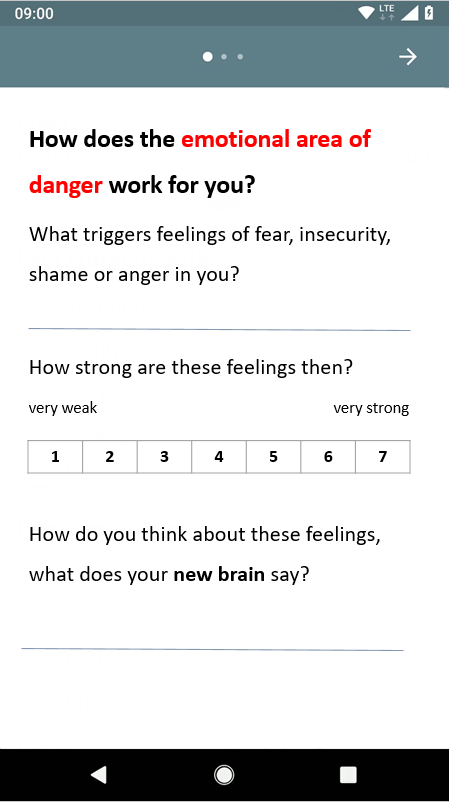
 *“Let's start with the emotion area of danger. How does this emotion area work for you? What triggers negative emotions for you, such as fear, insecurity, shame, or anger?* (Validate answer*) Let's fill that in, too! To do so, click on the text box and a keyboard will open. Next, please rate how strong these feelings are at such moments. On a scale from 1 (= very weak) to 7 (= very strong), how strong are your feelings then?* (Validate answer) *You can also enter this in the app - just select the number that suits you. Now let's consider how you think about these feelings. You remember that earlier we talked about the new brain, which is responsible for complex thinking and planning but also for self-criticism and worry. What does your new brain say in situations where you have these feelings?* (Validate answer) *Let's put that in too! Okay, done, now let's look at the next emotion area, please click on for that."*
- Work on the other two emotion areas in the same way, validate answers. If the participant cannot think of anything at first, make it clear that this is not a bad thing. Offer help, for example, think about the last time you had this feeling to get an example situation. Finally: „*Great, we've been thinking about your Emotional Compass together! It's absolutely okay if you found the exercise difficult or exhausting - a lot of people do."*
- *“With our Emotional Compass, when* ***RED t****akes control, our goal is to strengthen* ***GREEN****!* ***GREEN*** *calms* ***RED*** *and is a safe foundation for* ***BLUE****."* (click to continue)
- *“There are several ways to strengthen green to calm down and feel secure. One way is to learn to pay attention to your own breath. Shall we look at that together right now, too? All right, click the "Proceed to the Breathing Exercise!" button for that."*

### Breath counting

#### [
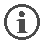
](https://www.google.com/url?sa=i&rct=j&q=&esrc=s&source=images&cd=&cad=rja&uact=8&ved=2ahUKEwj6iKDkjYLhAhUIDuwKHYniA0oQjRx6BAgBEAU&url=https://de.wikipedia.org/wiki/Datei:Infobox_info_icon.svg&psig=AOvVaw379mimxNArReUBgp9p_TUm&ust=1552669234895208)Information: Breath counting

- *“Great that you want to do a breathing exercise while you're at it! The breathing exercise can help you calm down. The goal of the exercise is for you to learn a way to find a* ***calming and soothing*** *breathing rhythm."* (click to continue)
- “*That means we focus on the green emotional area, the calm and security system, and aim to strengthen it.*" (click to continue)
- *“There are two options for the exercise now: You can listen to an audio file to guide you through the exercise, or you can read the exercise on your own. Please select which you prefer on the screen."* If audio is selected, the next instructions will be omitted and you will not restart until you complete the exercise.
- *“All right, you've decided to read through the exercise with me. As a first step, before we begin the breathing exercise, take some time to assume a* ***comfortable and upright posture****. You may choose to lie down, stand, or sit, whichever seems most comfortable for you at the time."* (If the participant wishes to sit, the therapist will also assume an upright and relaxed posture to set an example for the participant) (click to continue).
- *"Try to find a posture that makes you feel* ***confident and open****. Provided you have chosen to sit or stand, it may be helpful to stand with both soles of your feet flat on the floor and you try to straighten your shoulders and back at the same time."* (Therapist should demonstrate)
- *"Have you found a comfortable posture?"* (Assist as needed, emphasizing that any posture is okay as long as the person finds it comfortable. If the person can't decide, suggest if necessary that they try one posture, but of course can change it as the exercise progresses if it feels better another way) *"First take a few* ***deep, slow breaths****. Perhaps as you breathe deeply in and out, you will already recognize a rhythm that is comfortable for you?"* (short pause to breathe, click on).
- *"Try to notice how the* ***air slowly flows into and also out of your body****. Here, it can be helpful to focus on your nose to feel the air flowing through your nose."* (pause briefly to breathe, continue clicking)
- *"This can be* ***very difficult****! So it's perfectly okay if you find that your mind wanders off during the exercise. Then simply return your attention to the breath and* ***try not to view the digression as a mistake or judge it negatively.****"* (click to continue)
- *"Conscious breathing is like a muscle that you have to train. It is therefore perfectly normal if you find the exercise difficult at first. You're going to practice counting your breath right now. In doing so, you will learn to focus on your breath."* (click to continue)

#### [
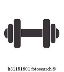
](https://www.google.com/url?sa=i&rct=j&q=&esrc=s&source=images&cd=&cad=rja&uact=8&ved=2ahUKEwiZj8uHjoLhAhXOy6QKHX8wAOYQjRx6BAgBEAU&url=https://www.fotosearch.de/clip-art/hantel.html&psig=AOvVaw1I-dWoT1U0d0S0a77ermuV&ust=1552669309693258) Exercise: Counting breath

- *"Now let's begin the breathing exercise. Please close your eyes right away and with each inhale and each exhale count internally to 5, with a pause in between that is comfortable for you. The entire exercise will take* ***3 minutes*** *and you will be alerted to the beginning and end of the exercise with a gong. Do you have any questions?* (Answer questions) *When you are ready to begin the exercise, press the button on the screen!"*


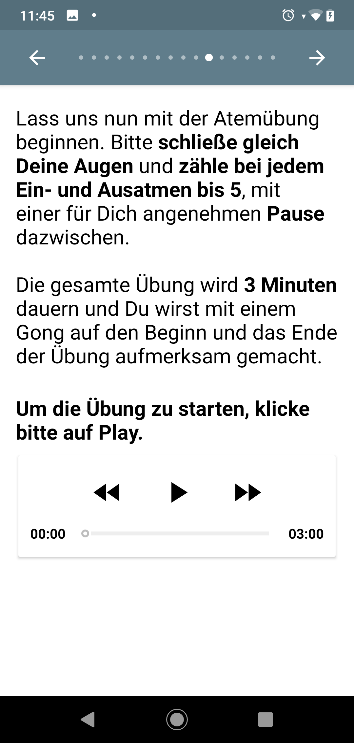

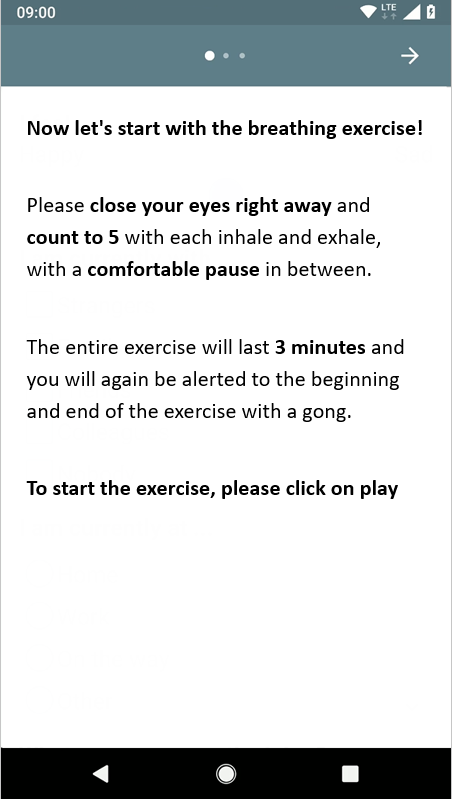


- *"I'm glad you did the breathing exercise. That's how you practiced* ***calming yourself down****. If that hasn't worked out so well yet, that's no problem, most people feel that way! Since our* ***everyday life*** *is usually* ***very hectic****, it is very difficult for us to stop for a few minutes and focus "only" on our breath."* (click more)
- *"No matter how it just worked out, you are on a good path. Through practice you will always get better! With practice, your breath can become your own personal* ***anchor of calm*** *that you always have with you in everyday life. How did you feel about the exercise?"* (Validate experiences of the participants, click on)

Explain gamification

- *"You will earn points from now on! You will get points when you think about your feelings in everyday life and when you do exercises. When your EMIcompass score is high enough, you will move up to the next level. So the more points you earn, the higher your EMIcompass level will be at the end."*

### *Closing of the session*

Praise for effort in lesson, clarify questions, summarize

- *"We really got a lot done today, that was certainly exhausting for you! You are on a very good path and have already worked a lot today on your self-care in dealing with feelings in everyday life. Do you have any questions about the exercises? What would be your conclusion to our session today, what do you take away?"*

Discuss organizational issues

- *"Finally, I would like to discuss with you what will happen next: You will take the cell phone home/to the ward today. So that you can consolidate what we have practiced together today, please practice independently over the next few days. The smartphone will remind you to practice once a day. However, you do not have to do the exercise from memory. The app will guide you, just as you have seen today. If you ever don't have time when the app reminds you to do your daily exercise, the app will remind you again at a later time. We can set here in the app when it is best for the smartphone to remind you to exercise. This setting will remain the same throughout the course of the study. What time do you have most days, when would you like to practice?"* (Open initial questionnaire in the app and select time - See DEMO printout for illustration).
- *"Also, you can allow us to ask you questions three days a week about your mood and situations you experience in your daily life. The app would then prompt you six times a day to briefly answer some questions about what you're doing and how you're feeling. This would have the advantage of allowing the app to provide you with an exercise exactly when there is a need. Based on our clinical experience and previous research findings, we believe that these small mood queries contribute to better treatment outcomes. You yourself get to decide whether or not you want us to set this option for you. Would you like to use the option?" 🡪* If the TN allows the queries: *from when and until when may we send you the mood queries?* (enter the times in the initial questionnaire, enter them in the Mutable Value Editor at Interactive 1🡪 see Technology Guide, use DEMO printout for illustration).
- *"If necessary, you can also do additional exercises on your own at any time; you don't have to wait specifically for the app to prompt you. So if you want to do additional exercises in between, open the app and select "Perform exercise", for example. Here you can then select which of the known exercises you would like to perform. Also, under "What have I learned so far?" you can look at all the known exercises again in detail with all the explanations."* (Make sure participants understand the difference, use DEMO printout for illustration)
- *"In exactly one week, something will change in your app. In addition to the exercises you already know, a new task will appear in the main menu of the app. The exercise will be explained to you via your smartphone. We won't see each other next week, so we will call you to hear how the exercises are going and to let you know that there is a new exercise. Or we can email you, in which case I would need an email address where I can reach you. What would you prefer?*

*It would be important that you do this exercise promptly then. At what time can we reach you well? If you have any questions or any other difficulties, you can also contact me or my colleagues on your own initiative. You can reach us by e-mail at* [*EMIcompass2@zi-mannheim.de*](mailto:EMIcompass2@zi-mannheim.de)*. You can also reach us by phone, we have set up an answering machine at 0621/ 1703-1934 where you can leave us a message. We will then call you back as soon as possible".*

- Make new appointment in two weeks, discuss procedure for cancelling appointments

## Between sessions (2nd week)

There will be no therapy session during this week, the participants will learn a new exercise on their own guided by the EMICOMPASS app. Exactly one week after the first session, the study therapist or the support staff (please make arrangements!) will contact the participants by phone at the agreed time or send an email (example under 6. Template for mail contact). Beforehand, the participants' data must be downloaded and reviewed so that you can give the participants feedback in this regard.

*How do I see what participants have completed and how many exercises they have done?*

Feedback on adherence should be provided by the support staff as a printout before the session - should be in the participant's folder, please check before the session!

### Topics for the telephone call

- How the participant is feeling
- Previous experience with the app, questions and difficulties
- Feedback on participant's adherence (positively reinforce good adherence; do not reprimand low adherence, emphasize importance and consider together how it could be better next week). To progress to the next week of intervention, participants must do at least the newly introduced exercise and one exercise to reinforce during the week. If these requirements are not met, participants remain in that week of intervention. If participants express great difficulty or desire to do so on their own, a week of intervention may also be repeated based on clinical impression. (For settings, see technique guide).
- Note that a new exercise (see 2. EMICOMPASS Intervention - Overview) should now appear in the app and that it should be done promptly.
- Remind of appointment in the following week

## Second session – Level 1 (3rd week)

### Overview of the second session – Level 1

|  |  |
| --- | --- |
| Introduction | 5 min |
| Debriefing of last week, decision for intervention arm | 10 min |
| [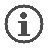](https://www.google.com/url?sa=i&rct=j&q=&esrc=s&source=images&cd=&cad=rja&uact=8&ved=2ahUKEwj6iKDkjYLhAhUIDuwKHYniA0oQjRx6BAgBEAU&url=https://de.wikipedia.org/wiki/Datei:Infobox_info_icon.svg&psig=AOvVaw379mimxNArReUBgp9p_TUm&ust=1552669234895208)Information: My calm, safe place | 5 min |
| [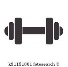](https://www.google.com/url?sa=i&rct=j&q=&esrc=s&source=images&cd=&cad=rja&uact=8&ved=2ahUKEwiZj8uHjoLhAhXOy6QKHX8wAOYQjRx6BAgBEAU&url=https://www.fotosearch.de/clip-art/hantel.html&psig=AOvVaw1I-dWoT1U0d0S0a77ermuV&ust=1552669309693258)Exercise: My calm, safe place | 20 min |
| Closing of the session | 10 min |

### Introduction

- Welcome participants, express joy about second appointment
- Ask how they are feeling
- Discuss agenda: *"First, I would like to know how the time has been for you since our last meeting and hear how the exercises are going. Also, I would love to do a new exercise with you so that you have even more tools in your daily life to help you feel calm and secure. Do you agree with this plan for our session today?"*

### Debriefing of last week, decision for intervention arm

- Last week's debrief: *"Since our last meeting, you first practiced counting your breath and thought about your emotional compass. Then last week you added a new exercise, you practiced imagining your one calming color. How did you do with the previous exercises?* (Ask more questions as needed for understanding until you feel you have a good gauge of which intervention arm participants would benefit better in. Include impression from first contact. (For overview of all exercises still to come, see 2. EMICOMPASS Intervention - Overview) *What did you find easy, where were these difficulties? Did you find the exercises helpful, which one helped you most? Were you able to visualize your color well? Were there any difficulties with the smartphone?"*
- Provide adherence feedback (adherence feedback should be provided as a printout by the support staff before the session - should be in the participant's folder, please check before the session!): *"I saw that you were very motivated and practiced a lot since our last meeting! That's great!"* If not practicing as much: *"I saw that unfortunately you haven't practiced very much since our last meeting. What was the reason for that?* (Showing understanding) *It would be important for them to practice a little more often next week, let's figure out together how to make that happen!"*
- If EMA was allowed, provide feedback on adherence here as well: *"I also noticed that you answered a lot of mood queries, great!"* for low adherence: *"I've seen that it's been difficult for you to answer the mood queries lately."*
- Decide in which intervention arm participants will benefit more, set appropriate exercise on smartphone (see technology guide)

### My calm, safe place

#### [
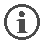
](https://www.google.com/url?sa=i&rct=j&q=&esrc=s&source=images&cd=&cad=rja&uact=8&ved=2ahUKEwj6iKDkjYLhAhUIDuwKHYniA0oQjRx6BAgBEAU&url=https://de.wikipedia.org/wiki/Datei:Infobox_info_icon.svg&psig=AOvVaw379mimxNArReUBgp9p_TUm&ust=1552669234895208) Information: My calm, safe place

Introduce new exercise:

- *"Today we want to learn a new exercise! The exercise is called "Finding a calm and safe place". Let's look at the exercise together! The exercise can help you* ***calm down and feel more secure*** *- much like the exercises before. In this exercise, you have the task of finding a place that is* ***calm and safe*** *for you. As you'll notice, this exercise is very similar to last week's exercise in which you practiced imagining a very personal color."* (click to continue)
- *"But before you find a* ***calm, safe place*** *for yourself, it can be helpful to regain a comfortable posture and find a breathing rhythm that is comfortable for you. Try to find a posture that makes you feel* ***confident and open****. Again, you can choose to sit, lie down or stand. If you choose to sit or stand, it may again be helpful for you to stand with both soles of your feet flat on the floor and you simultaneously try to* ***straighten your shoulders*** *and back."* (click to continue)

#### [
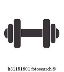
](https://www.google.com/url?sa=i&rct=j&q=&esrc=s&source=images&cd=&cad=rja&uact=8&ved=2ahUKEwiZj8uHjoLhAhXOy6QKHX8wAOYQjRx6BAgBEAU&url=https://www.fotosearch.de/clip-art/hantel.html&psig=AOvVaw1I-dWoT1U0d0S0a77ermuV&ust=1552669309693258) Exercise: My calm, safe place

- *"Let's start the exercise! Imagine a place that* ***calms*** ***you down and seems peaceful*** *to you. Remember that there is* ***no right or wrong place*** *- it's your own personal choice."* (click to continue)
- *"****Take your time*** *to decide and try to consciously observe what place or places come to mind. For example, it could be a place you have visited* ***many times in the past****. But you also have the possibility to imagine a completely* ***new place****. To do this,* ***close*** *your eyes for a moment!"* (Pause. Wait to see what comes to mind and encourage participants.) *"Have you thought of a place yet?"* (click to continue)
- If at first nothing comes to mind for the participant: *"Please* ***don't be frustrated*** *if you don't come up with a place right away - that's normal!* ***Take as much time as you need to search and decide****."* (click to continue)
- If they think of several places: "*It is also not bad if you think of several places at once and you can't decide directly which place you want to choose.* ***Take as much time as you need to search and decide.****"* (click to continue)
- *"If you have already decided on a place, you can now select "I have found my place". If you haven't found a place for yourself yet, that's no big deal at all. Just choose "I can't think of a place" and we'll think about it again together!"* (Participants make their selection)
- If "I can't think of a place" was selected: *"It's not bad that you haven't thought of a place yet, a lot of people feel that way. Feel free to take more time. It might help you to focus on your breath again. Maybe the short breathing exercise will help you to relax a bit before you start to search for your calm and safe place again without rushing. To do this, simply breathe in and out calmly, counting to 5 with a comfortable pause in between - just as you've been practicing for the past few weeks."* (do breathing exercise for 1-2 minutes, then click on next)
- *"If you have already thought of a place now, you can just keep clicking. If not, on the next page there is the option to select a place that is most closely associated with calm and safety for you. That place will then be your very own calm and safe place starting today!"*  (click to continue)
- If no place has been found yet*: "Just mentally choose the place here that is most likely to represent peace and security for you. Which one would you like to take?* (validate answer TN) *Okay, then click next."* (click to continue)
- *"Very nice, you have found your personal calm and safe place! In the exercise, you are to try to explore the place in your imagination. In doing so, try to imagine yourself in the place as vividly as possible."* (click to continue)
- *"It is perfectly okay if your place appears less clear in your mind's eye or disappears altogether. That is completely normal! Then simply return with your attention to your personal place, completely without negative evaluation."* (click to continue)
- *"Do you have any questions up to this point? If you are ready, we will then start with the actual imagining exercise! Go to your place in your mind right away. Try to focus on what you can see when you are in your personal place. This part of the exercise will take two minutes. As you know it from previous exercises, a gong will alert you to the beginning and end of the exercise. When you are ready, please click the button and close your eyes to begin the exercise!"*
- *"Great, you've explored your place for the first time! Now try to focus on other features of your place. Maybe you hear something, you see other people, or maybe you notice a sensation on your body, such as the warmth of the sun or a slight breeze. Let's take another two minutes so you can explore your personal place further! When you are ready, please start the exercise again with the button"*
- *"****Very nice****, you have found a* ***calm and safe place for yourself*** *today.* ***You pictured the place in your mind*** *and explored it! On a scale of 1 (very bad) to 7 (very good), how well were you able to imagine your safe place? (Record on paper sheet) How did you feel about the exercise?* (Praise, validate experience. If difficult, emphasize that many people have a hard time at first, that this is normal, etc.) *Your* ***safe place*** *can also become* ***your own personal anchor of calm and security*** *with a little practice. Just like your* ***breath,*** *you always have your imagination with you. So you can always call* ***your place to mind*** *- wherever you are and no matter how you're feeling right now."* (click to continue)


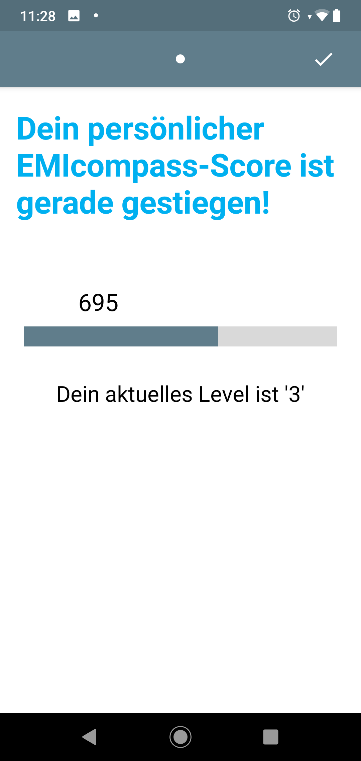

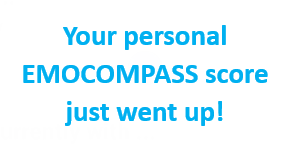

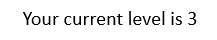


- *"Your EMICOMPASS score has continued to rise - great how many points you've already earned!"*

### *Closing of the session*

Praise for the effort in the lesson, clarify questions, summary

- *"We really got a lot done again today, which was certainly exhausting for you! You are on a very good path and have already worked a lot on your self-care in dealing with feelings in everyday life in the last few weeks. Do you have any questions about the exercises? What would be your conclusion to our session today, what do you take away? "*

Discuss organizational matters

- *"Finally, I would like to discuss with you again how to proceed. We will have our next appointment in two weeks* (make appointment). *In the meantime, you will again be reminded daily by the smartphone about your exercise - was the time okay, or should we change something here*? (Make changes? See tech guide) *If you ever don't have time when the app reminds you of your daily exercise, the app will remind you again at a later time. Of course, you can continue to do exercises on your own and don't have to wait for the signal from the app. A week from today, just like last week, you will again learn a new exercise on your own via the smartphone. It would be important that you then do this exercise again promptly. Again, we will call or mail you to let you know that there is a new exercise.*
- *“Also, you can continue to allow us to ask you questions three days a week about your mood and situations you experience in your daily life. The app would then prompt you six times a day to briefly answer some questions about what you're doing and how you're doing. If participants have had this in the last few weeks: it would continue exactly as you are used to. Would you be okay with that? If participants have not had it in the last few weeks: The app would then prompt you six times a day, three days a week, to briefly answer some questions about what you are doing and how you are doing. This would have the advantage that the app could offer you an exercise exactly when there is a need. In addition, it would also help researchers better understand what stresses young people in their daily lives. Based on our clinical experience and previous research findings, we believe that these small mood queries will help improve treatment outcomes. You yourself get to decide whether or not you want us to discontinue this option for you. Would you like to use the option?"* If yes*: "From when and until when may we send you mood surveys?"* (if participated in advance*) "Should we stay with the times we discussed?"* (change if sentiment queries are allowed or times 🡪 see tech guide)
- *"Is there anything else to discuss from your side, do you have any questions?"*
- Farewell

## Second session - Level 2 (3rd week)

### Overview of the second – Level 2

|  |  |
| --- | --- |
| Introduction | 5 min |
| Debriefing of last week, decision for intervention arm | 10 min |
| [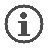](https://www.google.com/url?sa=i&rct=j&q=&esrc=s&source=images&cd=&cad=rja&uact=8&ved=2ahUKEwj6iKDkjYLhAhUIDuwKHYniA0oQjRx6BAgBEAU&url=https://de.wikipedia.org/wiki/Datei:Infobox_info_icon.svg&psig=AOvVaw379mimxNArReUBgp9p_TUm&ust=1552669234895208)Information: Compassionate companion | 10 min |
| [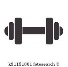](https://www.google.com/url?sa=i&rct=j&q=&esrc=s&source=images&cd=&cad=rja&uact=8&ved=2ahUKEwiZj8uHjoLhAhXOy6QKHX8wAOYQjRx6BAgBEAU&url=https://www.fotosearch.de/clip-art/hantel.html&psig=AOvVaw1I-dWoT1U0d0S0a77ermuV&ust=1552669309693258)Exercise: Compassionate companion | 15 min |
| Closing of the session | 10 min |

### Introduction

- Welcome participants, express joy about second appointment
- Ask how they are feeling
- Discuss agenda for session: *"First, I would like to know how the time has been for you since our last meeting and hear how the exercises are going. Also, I would love to do a new exercise with you so that you have even more tools in your daily life to help you feel calm and secure. Do you agree with this plan for our session today?"*

### Debriefing of last week, decision for intervention arm

- Debriefing from last week: *"Since our last meeting, you first practiced counting your breath and thought about your emotional compass. Then last week you added a new exercise. You practiced imagining your one calming color. How did you do with the exercises?* (If needed for understanding, ask more questions until you feel you have a good gauge of which intervention arm participants would benefit better in. Include impression from first contact) *What did you find easy, where were these difficulties? Did you find the exercises helpful, which one helped you the most? Were you able to visualize your color well? Were there any difficulties with the smartphone?"*
- Provide adherence feedback (adherence feedback should be provided as a printout by the support staff before the session - should be in the participant's folder, please check before the session!): *"I saw that you were very motivated and practiced a lot since our last meeting! That's great!"* If not practicing as much: *"I saw that unfortunately you haven't practiced very much since our last meeting. What was the reason for that?* (Showing understanding) *It would be important for them to practice a little more often next week, let's figure out together how to make that happen!"*
- If EMA was allowed, provide feedback on adherence here as well: *"I also noticed that you answered a lot of mood queries, great!"* for low adherence*: "I've seen that it's been difficult for you to answer the mood queries lately."*

### Compassionate companion

#### [
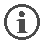
](https://www.google.com/url?sa=i&rct=j&q=&esrc=s&source=images&cd=&cad=rja&uact=8&ved=2ahUKEwj6iKDkjYLhAhUIDuwKHYniA0oQjRx6BAgBEAU&url=https://de.wikipedia.org/wiki/Datei:Infobox_info_icon.svg&psig=AOvVaw379mimxNArReUBgp9p_TUm&ust=1552669234895208)Information: Compassionate companion

Guide to the exercise:

- *"Today we want to learn a new exercise! The exercise is called "My compassionate companion". Let's look at the exercise together! The exercise can help you be more compassionate with yourself and feel secure in your everyday life- much like the exercises before."* (click to continue)
- *"But before we start the exercise, it can be helpful to regain a comfortable posture and find a* ***breathing rhythm that is comfortable*** *for you. Try to find a posture that makes you feel* ***confident and open****. Again, you can choose to sit, lie down or stand. If you choose to sit or stand, it may again be helpful for you to stand with* ***both soles of your feet flat on the floor*** *and you simultaneously try* ***to straighten your shoulders and back****."* (click to continue)

#### [
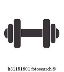
](https://www.google.com/url?sa=i&rct=j&q=&esrc=s&source=images&cd=&cad=rja&uact=8&ved=2ahUKEwiZj8uHjoLhAhXOy6QKHX8wAOYQjRx6BAgBEAU&url=https://www.fotosearch.de/clip-art/hantel.html&psig=AOvVaw1I-dWoT1U0d0S0a77ermuV&ust=1552669309693258)Exercise: Compassionate companion

- *"Let us begin the exercise! You now have the tasks of picturing a personal companion who conveys compassion to you. Your compassionate companion knows you and knows what you have been through in life.”* (click to continue)
- *"Your companion has certain characteristics: he or she is only there for you and wants to take care of you so that* ***you feel good and have no worries****. He or she does not judge you no matter what you think or feel. Your compassionate companion* ***understands your problems and accepts you as you are.****"* (click to continue)
- *"Your compassionate companion may be anything you can think of. It can be an* ***animal,*** *a* ***fantasy creature****, or even a* ***sun****. It can be a person you know or someone completely foreign."* (click to continue)
- *"You can choose your compassionate companion freely. Your compassionate companion can be old or young, male or female. Perhaps your compassionate companion has experienced something similar to you?"* (click to continue)
- *"Please close your eyes now and go in search of your compassionate companion. Take your time in doing so. If you cannot think of a companion, you could also focus on your* ***breath*** *again for a short time. Then turn back to the task of finding your* ***compassionate companion*** *in a* ***relaxed and non-judgmental*** *way."* (Wait, give participants time to find a compassionate companion). (click to continue)
- *Have you found your* ***compassionate companion****?*
  - YES: *"Great, then please select "I have found my compassionate companion" here"* (keep clicking)
  - NO: *„It's no problem if you haven't thought of a compassionate companion yet. Feel free to take your time with this and choose what feels good to you."* (click to continue)
- Only if NO was answered, *"When choosing your* ***compassionate companion****, it might help to think of someone you feel comfortable with. There is no right or wrong with this exercise either, just choose what comes to mind."* (Repeat compassionate companion characteristics again if needed.) (click to continue)
- *"****Great****, you have found your* ***compassionate companion****! It's perfectly okay if your* ***compassionate companion*** *appears less clearly in your mind's eye or disappears completely during the following exercise. Then simply imagine him again* ***without immediately judging this negatively****."* (click to continue)
- *"Now please* ***close your eyes*** *and take* ***2 minutes*** *to* ***picture your*** ***compassionate companion****. The beginning and the end of the exercise will again be signaled to you with a gong. When you are ready, please click the button and close your eyes to begin the exercise!"* (wait for exercise) (click on next)
- *"Very well done! Your* ***compassionate companion*** *can help you in difficult situations,* ***strengthening your calm and security system****."* (click to continue)
- *"Now, to practice doing just that, recall* ***a situation*** *that you experienced as* ***unpleasant****. To make it easier for you to remember, it is best to choose a current situation with other people in which you felt ashamed or anxious. Please do not choose a situation that is too bad. Can you think of a situation?"* (click YES or NO)
  -
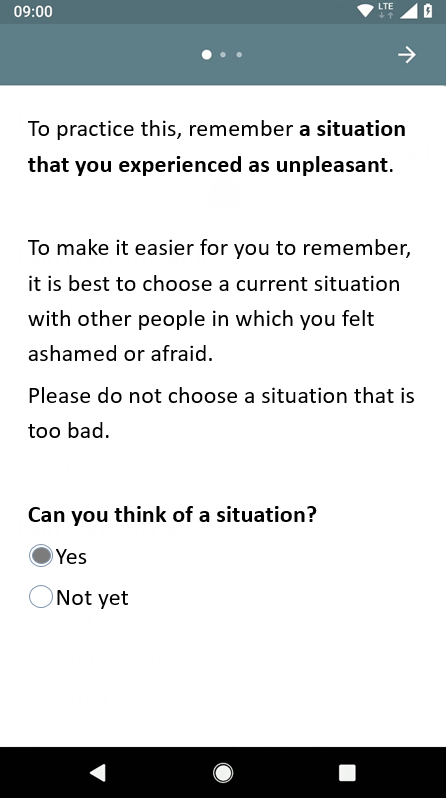
 YES: (have the situation told). *"How would you rate the situation on a scale from 1(= not bad at all) to 7 (= very bad)?"* (Make sure that participants choose unpleasant but not traumatic situations! Optimal difficulty between 3 and 6)
  - NO: "It's not bad if you can't think of a situation right away. Maybe you can remember a situation where you were rejected or excluded or a thing that embarrassed you. Maybe you can think of a situation where you were disappointed or other people were unkind to you. Can you think of anything?" If NO: "Feel free to take some more time and think about the last few days and weeks. If you thought of something, please select "I thought of a situation"! How would you rate the situation on a scale of 1(= not bad at all) to 7 (= very bad)?" (Make sure that participants choose unpleasant but not traumatic situations!!! Optimal difficulty between 3 and 6)
- *"Now please imagine this unpleasant situation pictorially for* ***2 minutes****. The beginning and the end of the exercise will again be signaled to you with a gong.* ***Please remember****: Where are you? What can you see or hear? Who else is there? What has happened? When you are ready, start the exercise by pressing the button. Then close your eyes and picture the situation."*
- *"****Very nice****! It must not have been easy to imagine this unpleasant situation again. On a scale of 1 (very low) to 7 (very high), how high is your tension right now (1-7)?"* (Write down on paper sheet)
- *"Your* ***compassionate companion*** *can now help you calm down after imagining this uncomfortable situation. Now take another* ***2 minutes*** *to imagine your* ***compassionate companion with your eyes closed****. When you are ready, start the exercise by clicking the button!"*
- *"On a scale of 1(very poor) to 7 (very good), how well were you able to visualize your compassionate companion? (Write down on paper sheet) How did you feel about the exercise?* (Praise, validate experience. If difficult, emphasize that many people find it difficult at first, that this is quite normal, etc.) *Your compassionate companion can also, with practice,* ***become your own personal anchor of calm and security****. Just like your* ***breath****, you always have your imagination with you. So* ***you can always call your compassionate companion to mind*** *- wherever you are and no matter how you're feeling right now."* (click to continue)
- *“Your EMICOMPASS score has continued to rise - great how many points you've already earned!"*

### *Closing of the session*

Praise for the effort in the lesson, clarify questions, summary

- *"We really got a lot done again today, that was certainly exhausting for you! You are on a very good path and have already worked a lot on your self-care in dealing with feelings in everyday life in the last few weeks. Do you have any questions about the exercises? What would be your conclusion to our session today, what do you take away? "*

Discuss organizational matters

- *"Finally, I would like to discuss with you again how to proceed. We will have our next appointment in two weeks* (schedule appointment)*. In the meantime, you will again be reminded daily by the smartphone about your exercise - was the time okay, or should we change something here?* (Make changes? See tech guide) *If you ever don't have time when the app reminds you of your daily exercise, the app will remind you again at a second time. Of course, you can continue to do exercises on your own and don't have to wait for the signal from the app. One week from today, just like last week, you will again learn a new exercise on your own via the smartphone. It would be important that you then do this exercise again promptly. Again, we will call or mail you to let you know that there is a new exercise.*
- *"Also, you can continue to allow us to ask you questions three days a week about your mood and situations you experience in your daily life. The app would then prompt you six times a day to briefly answer some questions about what you're doing and how you're doing."* If participants have had this in the last few weeks: *it would continue exactly as you are used to. Would you be okay with that?* If participants haven't had it in the last few weeks: *The app would then prompt you six times a day, three days a week, to briefly answer some questions about what you are doing and how you are doing This would have the advantage of allowing the app to provide you with an exercise exactly when there is a need. In addition, it would also help researchers better understand what stresses young people in their daily lives. Based on our clinical experience and previous research findings, we believe that these small mood queries will help improve treatment outcomes. You yourself get to decide whether or not you want us to discontinue this option for you. Would you like to use the option?"* If yes*: "From when and until when may we send you mood surveys?"* (if participated in advance) *"Should we stay with the times we discussed?"* (change if sentiment queries are allowed or times 🡪 see tech guide).
- *„Is there anything else to discuss from your side, do you have any questions?"*
- Farewell

## Between sessions (week 4)

There will be no therapy session during this week, the participants will learn a new exercise on their own guided by the EMICOMPASS app. Exactly one week after the first session, the study therapist or the support staff (please make arrangements!) will contact the participants by phone at the agreed time or send an email (example under 6. Template for mail contact). Beforehand, the participants' data must be downloaded and reviewed so that you can give the participants feedback in this regard.

### How do I see what participants have completed and how many exercises they have done?

Feedback on adherence should be provided by the support staff as a printout before the session - should be in the participant's folder, please check before the session!

### Topics for the phone call

- How the participant feels about the app
- Previous experience with the app, questions and difficulties
- Feedback on participant's adherence (positively reinforce good adherence; do not reprimand low adherence, emphasize importance and consider together how it could be better next week). To progress to the next week of intervention, participants must do at least the newly introduced exercise and one exercise to reinforce during the week. If these requirements are not met, participants remain in that week of intervention. If participants express great difficulty or desire to do so on their own, a week of intervention may also be repeated based on clinical impression. (For settings see technique guide)
- Note that a new exercise (see 2. EMICOMPASS Intervention - Overview) should now appear in the app and that it should be done promptly.
- Reminder of appointment in the following week

## Third session - Level 1 (5th week)

### Overview of the third session - Level 1

|  |  |
| --- | --- |
| Introduction | 5 min |
| Debriefing of the last week | 10 min |
| [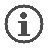](https://www.google.com/url?sa=i&rct=j&q=&esrc=s&source=images&cd=&cad=rja&uact=8&ved=2ahUKEwj6iKDkjYLhAhUIDuwKHYniA0oQjRx6BAgBEAU&url=https://de.wikipedia.org/wiki/Datei:Infobox_info_icon.svg&psig=AOvVaw379mimxNArReUBgp9p_TUm&ust=1552669234895208) Information: Surf the waves of your feelings | 5 min |
| [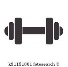](https://www.google.com/url?sa=i&rct=j&q=&esrc=s&source=images&cd=&cad=rja&uact=8&ved=2ahUKEwiZj8uHjoLhAhXOy6QKHX8wAOYQjRx6BAgBEAU&url=https://www.fotosearch.de/clip-art/hantel.html&psig=AOvVaw1I-dWoT1U0d0S0a77ermuV&ust=1552669309693258) Exercise: Surf the waves of your feelings | 20 min |
| Closure of the session | 10 min |

### Introduction

- Greet participants, express joy about third appointment
- Ask how you are feeling
- Discuss agenda for session: *"First, I would like to hear again how the time has been for you since our last meeting and hear how the exercises are going. Also, I would love to do a new exercise with you so that you have even more tools in your daily life to calm down and feel safe. Do you agree with this plan for our session today?"*

### Debriefing of the last week

- Last week's debrief: "*Since our last meeting, you first practiced imagining and exploring your calm and safe place. Then last week you added a new breathing exercise where you practiced breathing with pauses. How did you do with the exercises?* (Ask more questions for understanding if needed until you feel you have a good understanding of how the participant was doing and how to best support him/her) *What did you find easy, where were these difficulties? Did you find the exercises helpful, which one helped you the most? Do you have any questions about the exercises? Were there any difficulties with the smartphone?"*
- Provide adherence feedback (adherence feedback should be provided as a printout by the support staff prior to the session - should be in the TN's folder, please review prior to the session!): *"I saw that you were very motivated and practiced a lot since our last meeting! That's great!"* If not practicing as much: *"I saw that unfortunately you haven't practiced very much since our last meeting. What was the reason for that?* (Showing understanding) *It would be important for them to practice a little more often next week, let's figure out together how to make that happen!"*
- If EMA was allowed, give feedback on adherence here too: *"I also noticed that you answered a lot of mood queries, great!"* if adherence is low: *"I've seen that it's been difficult for you to answer the mood queries lately."*

### Surf the waves of your feelings

#### [
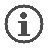
](https://www.google.com/url?sa=i&rct=j&q=&esrc=s&source=images&cd=&cad=rja&uact=8&ved=2ahUKEwj6iKDkjYLhAhUIDuwKHYniA0oQjRx6BAgBEAU&url=https://de.wikipedia.org/wiki/Datei:Infobox_info_icon.svg&psig=AOvVaw379mimxNArReUBgp9p_TUm&ust=1552669234895208)Information: Surf the waves of your feelings

Guide to the exercise:

- *"Also today we want to learn a new exercise! The exercise is called "Surf the waves of your feelings". Let's look at the exercise together! The exercise can help you be more compassionate with yourself and feel more secure in your everyday life - much like the exercises before."* (click to continue)

Inform about rational behind the exercise

- *"You can think about emotions in many different ways. One way would be to think of them as waves in the sea. In moments when we feel good, relaxed, and have little stress, our emotions resemble an ocean with shallow, small waves."*

*Before clicking on, little digression on feelings: Sometimes it's not so easy to identify what feeling you're having. Often we mix our feelings with what we feel in our body or with our thoughts. Sometimes we can tell if we are having a pleasant or unpleasant feeling but have a hard time naming it. What pleasant and unpleasant feelings do you know?* (Validate participant's answer, add if necessary if important feelings are missing. If the patient is very undifferentiated, spend a little more time here to differentiate this more precisely, then click on).

- *"Perhaps you have experienced it yourself, or maybe seen it in a movie or heard that the sea can change constantly? So can our emotions change quickly."* (click to continue)
- *"The sea can change greatly with an oncoming storm and suddenly make bigger waves. In our lives, too, many things can influence our emotions. For example, we might have problems at school, college, or work, or quarrels with family, so we don't feel so good and feel angry."* (click to continue)
- *"In the process, it can happen that we get carried away by the rising waves of emotions, they take us over completely and wash us to a place we didn't really want to be - we may even do or say things we didn't mean later. It's also possible that we run away from our emotions, ignore them, and don't take them seriously. Do you know anything like that about yourself?"*
- *"Today we are going to learn a new exercise that can help you perceive and feel emerging emotions more clearly. The goal is not to simply be carried away by the strong waves of emotions, but to surf on the waves that arise in life!"*

#### [
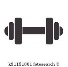
](https://www.google.com/url?sa=i&rct=j&q=&esrc=s&source=images&cd=&cad=rja&uact=8&ved=2ahUKEwiZj8uHjoLhAhXOy6QKHX8wAOYQjRx6BAgBEAU&url=https://www.fotosearch.de/clip-art/hantel.html&psig=AOvVaw1I-dWoT1U0d0S0a77ermuV&ust=1552669309693258) Exercise: Surf the waves of your feelings

- *"To practice surfing your emotions, please recall a situation that you experienced as unpleasant. To make it easier for you to remember, it is best to choose a current situation with other people in which you felt ashamed or anxious. Please do not choose a situation that is too bad. Can you think of a situation yet?"* (click YES or NO)
  - YES: (have the situation told). *"How would you rate the situation on a scale from 1(=not at all bad) to 7 (=very bad)?"* (Make sure participants choose unpleasant but not traumatic situations! Optimal difficulty between 3 and 6)
  - NO: *"It's not bad if you can't think of a situation right away. Maybe you can remember a situation where you were rejected or excluded or a thing that embarrassed you. Maybe you can think of a situation where you were disappointed or other people were unkind to you. Can you think of anything?"* If NO: *"Feel free to take some more time and think about the last few days and weeks. If you thought of something, please select "I thought of a situation"! How would you rate the situation on a scale of 1*(= not bad at all) *to 7* (= very bad)?" (Make sure that participants choose unpleasant but not traumatic situations!!! Optimal difficulty between 3 and 6)
- *"Now please imagine this unpleasant situation pictorially for* ***2 minutes****. The beginning and the end of the exercise will again be signaled to you with a gong.* ***Please remember:*** *Where are you? What can you see or hear? Who else is there? What has happened? When you are ready, start the exercise by pressing the button. Then close your eyes and picture the situation."* (click to continue)
- *“On a scale from 1 (very low) to 7 (very high), how high is your tension right now (1-7)?* (Write down on a sheet of paper) *Now try to perceive the emotions that have arisen because of the situation presented as clearly as possible and look at them closely. In doing so, try not to directly get rid of the emotions, block them, ignore them, or evaluate them negatively."* (Participants should be given the opportunity here to express their observations and be validated for them).
- *"Now try to look at your emotion like a wave that quickly gets bigger, has a peak, and then subsides. Allow yourself two minutes. The beginning and end of the exercise will again be signaled to you by a gong. When you are ready, start the exercise by pressing the button. Then close your eyes and imagine your emotions as waves in the ocean."*
- After the exercise: *"Very well done, that must not have been easy! It is not a problem if you found this exercise difficult. Many people feel that way. On a scale of 1* (very low) *to 7* (very high), *how high is your tension right now* (1-7)? (Write it down on a sheet of paper)" (click to continue)
- *"Remember, just like* ***a wave****, the emotions you are feeling right now, and have felt in the past, can also become less strong after a while - so they can come quickly and abruptly, but they can also go away."* (click to continue)
- *"Sometimes it can be helpful to* ***accept*** *the emotions you feel as they are. While our emotions are important, they don't make us as a person -* ***so we are not our emotions****."* (click to continue)
- *"That's why it's important to try not to hold on to our emotions for too long,* ***but also to let them go in order to realign our emotional compass****."* (click to continue)
- *"****Very nice****, you imagined your emotions as waves for the first time today! On a scale from 1 (very bad) to 7 (very good), how well were you able to imagine your emotions as waves? (Write down on paper sheet) How did you do with the exercise?* (Praise, validate experience. If difficult, emphasize that many people have a hard time at first, that this is normal, etc.) *This exercise can also help you calm down and deal better with your feelings. Like all the previous exercises, you can do this exercise anytime in your daily life, no matter where you are!"* (click to continue)
- *"Your EMICOMPASS score has continued to rise - great how many points you've already earned!"*

### Closing of the session

Praise for effort in lesson, clarify questions, summarize.

- *"We really got a lot done today, that was certainly exhausting for you! You are on a very good path and have already worked a lot today on your self-care in dealing with feelings in everyday life. Do you have any questions about the exercises? What would be your conclusion to our session today, what do you take away?"*

Discuss organizational matters

- *"In conclusion, I would like to discuss with you again how to proceed. This was the last appointment we had together to do exercises. In two weeks I would like to meet you again for a short final discussion to look back on the treatment together with you. After that you will also meet my colleague, Mrs. XXX, to do a final examination with her. Following this, you will then receive questions about your mood and situations you experience in everyday life via smartphone for another 4 days. The app will again prompt you six times a day to briefly answer some questions about what you are doing and how you are feeling. After these 4 days, please return the cell phone to us or send it to us with the return envelope, an agreement on this will be made in the final meeting with my colleague.* (An agreement should be made in the final diagnostic meeting and, if necessary, a return date should be agreed upon).
- *"Until two weeks from now, everything will run as usual: You will again be reminded of your exercise every day by the smartphone - was the time okay, or should we change something here?* (Make changes? 🡪 see Technology guide) *If you sometimes don't have time when the app reminds you of your daily exercise, the app will remind you again at a second time. Of course, you can continue to do exercises on your own and don't have to wait for the signal from the app. One week from today, just like last week, something will change again on your smartphone. Something new will appear again. It would be important for you to do this exercise promptly again then. Again, we will call or email you to let you know that there is a new exercise now."*
- *"Also, you can continue to allow us to ask you questions three days a week about your mood and situations you experience in your daily life. The app would then prompt you six times a day to briefly answer some questions about what you're doing and how you're doing."* If participants have had this in the last few weeks: *it would continue exactly as you are used to. Would you be okay with that?* If participants haven't had it in the last few weeks: *The app would then ask you six times a day, three days a week, to briefly answer some questions about what you are doing and how you are doing. This would have the advantage that the app can offer you an exercise exactly when there is a need. In addition, it would also help researchers better understand what stresses young people in their daily lives. Based on our clinical experience and previous research findings, we believe that these small mood queries will help improve treatment outcomes. You yourself get to decide whether or not you want us to discontinue this option for you. Would you like to use the option?"* If yes: *"From when and until when may we send you mood surveys?"* (if participated in advance) *"Should we stay with the times we discussed?"* (change if sentiment queries are allowed or times 🡪 see tech guide).
- *"Is there anything else to discuss from your side, do you have any questions?"*
- Farewell

## Third session - Level 2 (5th week)

### Overview of the third session - Level 2

|  |  |
| --- | --- |
| Introduction | 5 min |
| Debriefing of the last week | 10 min |
| [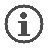](https://www.google.com/url?sa=i&rct=j&q=&esrc=s&source=images&cd=&cad=rja&uact=8&ved=2ahUKEwj6iKDkjYLhAhUIDuwKHYniA0oQjRx6BAgBEAU&url=https://de.wikipedia.org/wiki/Datei:Infobox_info_icon.svg&psig=AOvVaw379mimxNArReUBgp9p_TUm&ust=1552669234895208) Information: My compassionate Self | 5 min |
| [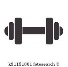](https://www.google.com/url?sa=i&rct=j&q=&esrc=s&source=images&cd=&cad=rja&uact=8&ved=2ahUKEwiZj8uHjoLhAhXOy6QKHX8wAOYQjRx6BAgBEAU&url=https://www.fotosearch.de/clip-art/hantel.html&psig=AOvVaw1I-dWoT1U0d0S0a77ermuV&ust=1552669309693258) Exercise: My Compassionate Self | 20 min |
| Closing of the session | 10 min |

### Introduction

- Greet participants, express joy about third appointment
- Ask how you are feeling
- Discuss agenda for session: *"First, I would like to hear again how the time has been for you since our last meeting and hear how the exercises are going. Also, I would love to do a new exercise with you so that you have even more tools in your daily life to calm down and feel safe. Do you agree with this plan for our session today?"*

### Debriefing of the last week

- Debriefing from last week: *"Since our last meeting, you first practiced picturing your compassionate companion with its different characteristics. Then last week you added a new exercise where you found a safe place for yourself and explored it. How did you feel about the* *exercises?* (Ask more questions for understanding if needed, until you feel you have a good understanding of how the participant was doing and how he/she can be optimally supported) *What did you find easy, where were there difficulties? Did you find the exercises helpful, which one helped you the most? Do you have any questions about the exercises? Were there any difficulties with the smartphone?"*
- Provide adherence feedback (adherence feedback should be provided as a printout by the support staff prior to the session - should be in the TN's folder, please review prior to the session!): *"I saw that you were very motivated and practiced a lot since our last meeting! That's great!"* If not practicing as much: *"I saw that unfortunately you haven't practiced very much since our last meeting. What was the reason for that?* (Showing understanding) *It would be important for them to practice a little more often next week, let's figure out together how to make that happen!"*
- If EMA was allowed, give feedback on adherence here too: *"I also noticed that you answered a lot of mood queries, great!"* if adherence is low: *"I've seen that it's been difficult for you to answer the mood queries lately."*

### My compassionate self

#### [
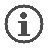
](https://www.google.com/url?sa=i&rct=j&q=&esrc=s&source=images&cd=&cad=rja&uact=8&ved=2ahUKEwj6iKDkjYLhAhUIDuwKHYniA0oQjRx6BAgBEAU&url=https://de.wikipedia.org/wiki/Datei:Infobox_info_icon.svg&psig=AOvVaw379mimxNArReUBgp9p_TUm&ust=1552669234895208)Information: My compassionate self

Guide to the exercise:

- *"Today we want to learn a new exercise! The exercise is called "My Compassionate Self". Let's look at the exercise together! The exercise can help you be more compassionate with yourself and feel secure in your everyday life- much like the exercises before."* (click to continue)
- *"Over the past few weeks, you have practiced using your imagination to access your* ***compassionate companion*** *in everyday life and to imagine your* ***safe place****. Although this has definitely been difficult and possibly exhausting for you at times, you have done everything very well so far! Keep up the good work!"* (click to continue)

Inform about rational behind the exercise

- *"Also today you will learn a new exercise that can help you calm yourself down and be more caring with yourself. In this way, we will again strengthen the green area of your emotional compass, the calm and secure system."* (click to continue)
- *"In the next exercise, you will learn to imagine your* ***compassionate self****. This will give you the opportunity to learn, step by step, to* ***be more caring and compassionate with yourself****. Your compassionate self can help you* ***feel more secure in your everyday life****."* (click to continue)
- *"But before we begin the exercise, it may be helpful to return to a* ***comfortable posture*** *and find a* ***breathing rhythm*** ***that is comfortable*** *for you. Try to find a posture that makes you feel* ***confident and open****. Again, you can choose to sit, lie down or stand. If you choose to sit or stand, it may again be helpful for* ***you to stand with both soles of your feet flat on the floor*** *and you simultaneously try* ***to straighten your shoulders and back****."* (click to continue)

#### [
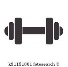
](https://www.google.com/url?sa=i&rct=j&q=&esrc=s&source=images&cd=&cad=rja&uact=8&ved=2ahUKEwiZj8uHjoLhAhXOy6QKHX8wAOYQjRx6BAgBEAU&url=https://www.fotosearch.de/clip-art/hantel.html&psig=AOvVaw1I-dWoT1U0d0S0a77ermuV&ust=1552669309693258) Exercise: My compassionate self

- *"The exercise has similarities to your compassionate companion. Your compassionate self has certain characteristics: just like your compassionate companion, your compassionate self* ***does not judge*** *you. It is* ***loving, warm and understands*** *you. It is* ***full of warmth and understanding****."* (click to continue)
- *"Your compassionate self can look like your reflection, or it can look like you in the future or in the past."* (click to continue)
- *"As you already know, there is no right or wrong in this exercise. It's your own personal choice how you want to envision your compassionate self."* (click to continue)
- *"As you do the exercise, imagine right now how you walk step by step toward your compassionate self and you become friendly and one. Imagine seeing the world through the eyes of your compassionate self and also seeing yourself compassionately. You do not judge and are loving and warm. You are full of warmth and understanding."* (click to continue)
- *"It's perfectly okay if you don't always have a clear picture in your mind's eye during the following exercise, or if the idea sometimes disappears altogether. Then simply return to the exercise mentally,* ***without any negative evaluation*** *at all!"* (click to continue)
- *"Please close your eyes right now and take two minutes to figuratively imagine looking at the world and yourself through the eyes of your compassionate self. As usual, the beginning and end of the exercise will be signaled with a gong. When you are ready, start the exercise by pressing the button."*
- After the exercise: *"Very nice! Your compassionate self can help you calm down in everyday life. The exercise can support you in becoming more caring and compassionate with yourself step by step in your everyday life as well. It's absolutely okay if you've sometimes had a hard time picturing your compassionate self and his/her compassionate view of the world - many people do!"* (click to continue)
- *"You always have the opportunity to think of your compassionate self wherever you are and no matter how you are feeling. Your compassionate self can be your personal anchor of peace and security."* (click to continue)
- ***"Very nice****, you have imagined your compassionate self for the first time today! On a scale of 1* (very bad) *to 7* (very good), *how well were you able to imagine your compassionate companion?* (Record on paper sheet) *How did you feel about the exercise?* (Praise, validate experience. If difficult, emphasize that many people have a hard time at first, that this is normal, etc.).
- *"Your EMICOMPASS score has continued to rise - great how many points you've already earned!"*

### Closing of the session

Praise for effort in lesson, clarify questions, summarize.

- *“We really got a lot done again today, that was certainly exhausting for you! You are on a very good path and have already worked a lot today on your self-care in dealing with feelings in everyday life. Do you have any questions about the exercises? What would be your conclusion to our session today, what do you take away? "*

Discuss organizational matters

- *"In conclusion, I would like to discuss with you again how to proceed. This was the last appointment we had together to do exercises. In two weeks I would like to meet you again for a short final discussion to look back on the treatment together with you. After that you will also meet my colleague, Mrs. XXX, to do a final examination with her. Following this, you will then receive questions about your mood and situations you experience in everyday life via smartphone for another 4 days. The app will again prompt you six times a day to briefly answer some questions about what you are doing and how you are feeling. After these 4 days, please return the cell phone to us or send it to us with the return envelope, an agreement on this will be made in the final meeting with my colleague.* (An agreement should be made in the final diagnostic meeting and, if necessary, a return date should be agreed upon).
- *"Until two weeks from now, everything will run as usual: You will again be reminded of your exercise every day by the smartphone - was the time okay, or should we change something here?* (Make changes? 🡪 see tech guide) *If you sometimes don't have time when the app reminds you of your daily exercise, the app will remind you again at a second time. Of course, you can continue to do exercises on your own and don't have to wait for the signal from the app. A week from today, just like last week, something will change again on your smartphone. Something new will appear again, building on today's exercise. It would be important for you to do this exercise then again in a timely manner. Again, we will call or email you to let you know that there is now a new exercise - was the time okay last time, or should we change something here?”*
- *"Also, you can continue to allow us to ask you questions three days a week about your mood and situations you experience in your daily life. The app would then prompt you six times a day to briefly answer some questions about what you're doing and how you're doing."* If participants have had this in the last few weeks: *it would continue exactly as you are used to. Would you be okay with that*? If participants haven't had it in the last few weeks: *The app would then prompt you six times a day, three days a week, to briefly answer some questions about what you are doing and how you are doing. This would have the advantage that the app could offer you an exercise exactly when there is a need. In addition, it would also help researchers better understand what stresses young people in their daily lives. Based on our clinical experience and previous research findings, we believe that these small mood queries will help improve treatment outcomes. You yourself get to decide whether or not you want us to discontinue this option for you. Would you like to use the option?"* If yes: *"From when and until when may we send you mood surveys?"* (if participated in advance) *"Should we stay with the times we discussed?"* (change if sentiment queries are allowed or times 🡪 see tech guide).
- *"Is there anything else to discuss from your side, do you have any questions?"*
- Farewell

## Between sessions (6th week)

There will be no therapy session during this week, the participants will learn a new exercise on their own guided by the EMICOMPASS app. Exactly one week after the first session, the study therapist or the support staff (please make arrangements!) will contact the participants by phone at the agreed time or send an email (example under 6. Template for mail contact). Beforehand, the participants' data must be downloaded and reviewed so that you can give the participants feedback in this regard.

### How do I see what participants have completed and how many exercises they have done?

Feedback on adherence should be provided by the support staff as a printout before the session - should be in the participant's folder, please check before the session!

### Topics for the telephone call

- How the participant is feeling
- Previous experience with the app, questions and difficulties
- Feedback on participant's adherence (positively reinforce good adherence; do not reprimand low adherence, emphasize importance and consider together how it could be better next week). To progress to the next week of intervention, participants must do at least the newly introduced exercise and one exercise to reinforce during the week. If these requirements are not met, participants remain in that week of intervention. If participants express great difficulty or desire to do so on their own, a week of intervention may also be repeated based on clinical impression. (For settings see technique guide)
- Note that a new exercise (see 2. EMICOMPASS Intervention - Overview) should now appear in the app and that it should be done promptly.
- Reminder of appointment in the following week

## Closing session

### Overview of the closing session

|  |  |
| --- | --- |
| Introduction | 5 min |
| Debriefing of the last week | 10 min |
| Review of the treatment | 10 min |

### Introduction

- Greet participants, express joy about last appointment
- Ask how you are feeling
- Discuss agenda for session: *"First, I would like to hear again how the time has been for you since our last meeting and hear how the exercises are going. Also, I would very much like to review the treatment with you today and end by giving you an overview of the exercises you learned during treatment. Do you agree with this plan for our session today?"*

### Debriefing of the last week

- Debriefing of last week for Level 1: *"Since our last meeting, you first practiced imagining your emotions as a wave and surfing on it, so to speak. Then last week, a summary of the exercises you learned during the treatment was added, you took a closer look at your toolbox, so to speak, and continued to practice these exercises. How did you do with the exercises?* (Ask more questions for understanding if needed, until you feel you have a good understanding of how the participant was doing and how he/she can be optimally supported) *What did you find easy, where were these difficulties? Did you find the exercises helpful, which one helped you the most? Do you have any questions about the exercises? Were there any difficulties with the smartphone?"*
- Last week's debrief for Level 2: *"Since our last meeting, you first practiced imagining your compassionate self and looking at the world and yourself through his/her eyes. Then last week you practiced writing a compassionate message to yourself. How did you do with the exercises?* (ask more questions for understanding if needed, until you feel you have a good understanding of how the participant was doing and how he/she can be optimally supported) *What did you find easy, where were these difficulties? Did you find the exercises helpful, which one helped you the most? Do you have any questions about the exercises? Were there any difficulties with the smartphone?"*
- Provide adherence feedback (adherence feedback should be provided as a printout by the support staff before the session - should be in the participant's folder, please check before the session!): *"I saw that you were very motivated and practiced a lot since our last meeting! That's great!"* If not practicing as much: "*I saw that unfortunately you haven't practiced very much since our last meeting. What was the reason for that?* (Showing understanding) *It would be important for them to practice a little more often next week, let's figure out together how to make that happen!"*
- If EMA was allowed, provide feedback on adherence here as well: *"I also noticed that you answered a lot of mood queries, great!" for low adherence: "I've seen that it's been difficult for you to answer the mood queries lately."*

### Review of the treatment

- *"To conclude, I think it would be nice if we could take a look back together! How was the treatment for you? Do you have any questions? What exercises did you find helpful? What do you take away?"*

Finally, say goodbye to participants, give them the participant manual and remind them of the diagnostic appointments!

# Potential difficulties in therapy

Potential difficulties in therapy could be low adherence and unreliability regarding appointments. It is important here that the therapeutic attitude remains approachable, empathetic and compassionate. Together with the participants, it should then be worked out how adherence or reliability can be improved. If necessary, there is also the possibility of remaining in one week of the intervention and repeating it rather than progressing to the next week of intervention if, for example, the new exercise has not been done at all. This should be decided on a case-by-case basis and together with the participants.

It is also conceivable that participants may devalue themselves during the session, for example, because they found it difficult to perform an exercise. At this point it is important to normalize difficulties with the exercises and to activate the participants' system of calm and security by dealing with their problems in an empathic and compassionate way.

In addition, self-compassion could be experienced as aversive, here especially information from the biographical anamnesis should be considered, as this is often necessary to be able to correctly classify the current behaviour of the participants.

For all difficulties it is true that these can and should be discussed in the supervision sessions.

**Deviations from the protocol:**

- Appointment with participants takes place a few days after the actual appointment:

Participants can continue to follow the intervention process. Work on a new exercise in the session. If this has already been done independently by the participants, it can be called up again via "What I have learned so far".

- Dates are delayed longer (e.g., because participants want to travel spontaneously).

The start of the intervention can be up to 4 weeks after randomization, so one option is to start the intervention after the trip. The intervention can also be paused for the time of the trip. Participants should hand in the smartphone in the meantime. After the break, a session takes place in which the participants receive the smartphone and re-join the intervention; if necessary, the session duration can be extended somewhat in order to refresh the content previously worked on.

In such cases, please consult with the study leader!

# Mail Contact Template

Dear XXX / Dear XXX,

Exactly one week ago we had our last session. Since we will not meet again until next week, I am writing you this mail to ask how you are doing and to give you feedback for the last week.

(With satisfactory adherence) From what I could see, you were very motivated in the meantime and used the app a lot! You practiced X times and answered X mood queries. That's impressive, keep it up!

(With poor adherence) From what I could see, it was difficult for you to use the app in the last few days. Maybe you just had a lot going on the last few days? That's okay, just try to start using the app more regularly again starting today. Remember, the exercises can help you calm down and feel secure even in difficult situations. You can also always write to us using the app if you have any questions or if something seems unclear/too difficult.

A new exercise will soon appear in the app, it is called "XXXX". It would be nice if you do the new exercise as soon as you have time. This is the only way to unlock new content and try out exciting new exercises.

If you have any technical difficulties or questions about the exercises, you can reach me by replying to this mail, writing to us directly through the app, or leaving me a message on the study answering machine (0621- 1703 1934).

Best regards

XXXXXX

P.S.: As a reminder, our next meeting will be on XX.XX.XXXX at XX o'clock room XXX!

# Supervision

Regular supervision sessions are planned as part of the study (every 2 weeks at the beginning, then every 4 weeks during the course). These will take place via video telephony and are to be prepared and followed up by the participants. The supervision protocol on the following page serves this purpose.

**Supervision protocol EMIcompass**

| **Date:** |
| --- |
| **Patient ID:** |
| **My concern(s) for the supervision + info on the adherence of the participant.** |
| **Notes taken during the supervision session:** |
| **Results/suggestions of the analysis of audio recordings:** |
| **Summary of key aspects of today's supervision/ What will I implement?:** |
