# Supplementary material for: A Hybrid Ecological Momentary Compassion–Focused Intervention for Enhancing Resilience in Help-Seeking Young People: Prospective Study of Baseline Characteristics in the EMIcompass Trial
Source: JMIR Form Res. 2022 Nov 4;6(11):e39511. doi: 10.2196/39511 (PMC9675017; doi:10.2196/39511)
Supplement: Multimedia Appendix 4 [file formative_v6i11e39511_app4.docx]

# Multimedia Appendix 4 – Modified criteria of the clinical staging model

**Table S1**. Inclusion criteria and transdiagnostic sample characteristics based on a modified version of the clinical staging model [5] as published in Schick, et al. [7].

| **Stage** | | **Criteria** | **Measure** |
| --- | --- | --- | --- |
| **1a (Distressed individuals)** | | Psychological distress (K10^a^ score ≥ 20) but not fulfilling criteria of stage 1b or 2 | K10 |
| **1b (CHARMS^b^)** | |  |  |
|  | Psychosis Trait Vulnerability | 1st degree relative with psychosis *and* SOFAS^c^ < 50 in the last 12 months  *or* SOFAS 30% below past level | Family risk  SOFAS |
|  | Psychosis Trait Vulnerability | Schizotypal personality *and* SOFAS < 50 in the last 12 months  *or* SOFAS 30% below past level | SCID II  SOFAS |
|  | Bipolar Trait Vulnerability | Depressed mood or diminished interest or pleasure for at least 1 week as well as 2 additional criteria of depression:   - weight loss, - sleep disorder, - psychomotor disturbances, - loss of energy, - feelings of worthlessness or guilt, - diminished ability to think or concentrate or indecisiveness, - suicidality   *And* mood swings for at least 6 months in the lifetime (not symptom-free for a longer time period than 2 months consecutively) and at least 3 symptoms:   - decreased need for sleep, - increased energy, - inflated self-esteem or grandiosity, - increase in goal-directed activity, - restlessness, - increased talkativeness, - unusual ideas, - risky behaviour, - inappropriate humour (does not have to equal loss of function!)   *Or* 1st degree relative with bipolar disorder | SCID-5^d^  Family risk |
|  | Attenuated Psychotic Symptoms | CAARMS global rating 3-6 *and* frequency of 3-6 on the subscales:   1. unusual thought content, 2. non bizarre ideas, 3. perceptual abnormalities, 4. disorganized speech   *Or:* global rating score of 6 *and* frequency of 3 on the subscales:   1. unusual thought content, 2. non bizarre ideas, 3. perceptual abnormalities, 4. disorganized speech | CAARMS^e^ |
|  | Attenuated Hypomanic Symptoms | Elevated, expansive or unusually irritable mood on at least 2 consecutive days *and*  2 (or in case of only irritable mood 3) additional criteria:   - inflated self-esteem or grandiosity, - decreased need for sleep, - increased talkativeness, - flight of ideas or subjective experience that thoughts are racing, - distractibility, - increase in goal-directed activity or psychomotor agitation, - unusual ideas, - increased involvement in activities, that are pleasurable in short-time but have a high potential for long-term damage   for a duration of max. 3 days, if 3 or more (or in case of only irritable mood 4 or more) additional criteria are met *and* there are functional disturbances *or* others notice the mood or functional disturbances  for a duration of max. 6 days, if 3 or more(or in case of only irritable mood 4 or more) additional criteria are met *or* there are functional disturbances or others notice the mood or functional disturbances  Exclusion: hospitalization, severe impairment in social or professional functioning, no psychotic elements | SCID-5 |
|  | Moderate (Attenuated) depression | SKID: mild or moderate depression (current or lifetime)  At least 1 cardinal symptom, 5 additional symptoms  *And* HAM-D > 17 (cutoff) | SCID-5  HAM-D^f^ |
|  | BLIPS^g^ | Global rating of 6 on the subscales   1. unusual thought content 2. non bizarre ideas   Global rating of 5 or 6 on the subscale perceptual abnormalities  *And/or* global rating of 6 on the subscale disorganized speech present for less than a week  *And* frequency: 4 - 6 on all above-named scales | CAARMS |
|  | Anxiety | SKID: mild - moderate panic disorder /agoraphobia (current or lifetime) *or*  SKID: not meeting criteria for GAS^i^, i.e., symptoms for less than 6 months or less than 4 symptoms met or  Diagnosis of a mild -moderate social phobia (current or lifetime)  *And* HAM-A > 9 (cutoff) | SCID-5  HAM-A^h^ |
| **2 (first treated episode)** | |  |  |
|  |  | Psychosis | CAARMS |
|  |  | Severe major depression (current or lifetime) | SCID-5 |
|  |  | Mania/ Hypomania | SCID-5 |
|  |  | Severe anxiety disorder (current or lifetime) e.g. agoraphobia, GAS | SCID-5 |

^a^Kessler Distress Scale (K10[6])

^b^Clinical High At-Risk Mental State (CHARMS)

^c^Social and Occupational Functioning Assessment Scale (SOFAS[2])

^d^Structured Clinical Interview for DSM-5 (SCID-5[1])

^e^Comprehensive Assessment of At-Risk Mental State (CAARMS[8])

^f^Hamilton Depression Rating Scale (HAM-D[4])

^g^Brief Limited Intermittent Psychotic Symptoms (BLIPS)

^h^Hamilton Anxiety Rating Scale (HAM-A[3])

^i^Generalized Anxiety Disorder (GAD).

In deviation to the clinical staging model proposed by Hartmann, et al. [5], we did not include participants from attenuated symptoms (stage 1b) or a first treated episode of borderline personality disorder. We added participants with stage 1b or stage 2 of anxiety disorders. In addition, we used a different clinician rated instrument to assess attenuated depression.

**References**

1. First MB, Williams JB, Karg RS, Spitzer RL (2015) Structured clinical interview for DSM-5—Research version (SCID-5 for DSM-5, research version; SCID-5-RV). American Psychiatric Association, Arlington, VA

2. Goldman HH, Skodol AE, Lave TR (1992) Revising axis V for DSM-IV: A review of measures of social functioning. Am J Psychiatry 149:9

3. Hamilton M (1959) The assessment of anxiety states by rating. British journal of medical psychology

4. Hamilton M (1960) A rating scale for depression. Journal of neurology, neurosurgery, psychiatry Res 23:56

5. Hartmann JA, Nelson B, Spooner R, Paul Amminger G, Chanen A, Davey CG, McHugh M, Ratheesh A, Treen D, Yuen HP, McGorry PD (2019) Broad clinical high-risk mental state (CHARMS): Methodology of a cohort study validating criteria for pluripotent risk. Early Interv Psychiatry 13:379-386

6. Kessler RC, Andrews G, Colpe LJ, Hiripi E, Mroczek DK, Normand SL, Walters EE, Zaslavsky AM (2002) Short screening scales to monitor population prevalences and trends in non-specific psychological distress. Psychol Med 32:959-976

7. Schick A, Paetzold I, Rauschenberg C, Hirjak D, Banaschewski T, Meyer-Lindenberg A, Boehnke JR, Boecking B, Reininghaus U (2021) Effects of a Novel, Transdiagnostic, Hybrid Ecological Momentary Intervention for Improving Resilience in Youth (EMIcompass): Protocol for an Exploratory Randomized Controlled Trial. JMIR Res Protoc 10:e27462

8. Yung AR, Yuen HP, McGorry PD, Phillips LJ, Kelly D, Dell 'olio M, Francey SM, Cosgrave EM, Killackey E, Stanford C, Godfrey K, Buckby J (2005) Mapping the onset of psychosis: the Comprehensive Assessment of At-Risk Mental States. Australian and New Zealand Journal of Psychiatry Research Assistant; Margaret Dell'Olio, Research Assistant; Shona M. Francey, Clinical Coordinator; Elizabeth M. Cosgrave, Research Fellow Research Fellow Research Assistant Research Assistant 39:964-971
