# Supplementary material for: A Hybrid Ecological Momentary Compassion–Focused Intervention for Enhancing Resilience in Help-Seeking Young People: Prospective Study of Baseline Characteristics in the EMIcompass Trial
Source: JMIR Form Res. 2022 Nov 4;6(11):e39511. doi: 10.2196/39511 (PMC9675017; doi:10.2196/39511)
Supplement: Multimedia Appendix 5 [file formative_v6i11e39511_app5.docx]

# Multimedia Appendix 5 – Correlation table

**Table S2.** Correlation table.

|  | **Clinical stage** | **Age** | **Gender** | **Minority**  **status** | **K10 ^a^** | **BSI ^b^** | **SOFAS** | **Momentary SC ^c^** | **Overall SC** | **Adaptive emotion regulation** | **Maladaptive emotion regulation** | **Training frequency** | **WAIP ^d^** | **WAIT ^e^** |
| --- | --- | --- | --- | --- | --- | --- | --- | --- | --- | --- | --- | --- | --- | --- |
| **Clinical stage** | 1.00 |  |  |  |  |  |  |  |  |  |  |  |  |  |
| **Age** | 0.00  *P*=.988 | 1.00 |  |  |  |  |  |  |  |  |  |  |  |  |
| **Gender** | 0.18  *P*=.244 | -0.04  *P*=.778 | 1.00 |  |  |  |  |  |  |  |  |  |  |  |
| **Minority status** | 0.17  *P*=.097 | 0.01  *P*=.935 | 0.07  *P*=.633 | 1.00 |  |  |  |  |  |  |  |  |  |  |
| **K10** | 0.38  *P*=.009 | -0.16  *P*=.293 | 0.21  *P*=.159 | 0.07  *P*=.655 | 1.00 |  |  |  |  |  |  |  |  |  |
| **BSI** | 0.14  *P*=.356 | -0.23  *P*=.117 | 0.07  *P*=.634 | -0.13  *P*=.377 | 0.77  *P*<.001 | 1.00 |  |  |  |  |  |  |  |  |
| **SOFAS ^c^** | -0.39  *P*=.008 | -0.06  *P*=.693 | -0.01  *P*=.943 | -0.15  *P*=.312 | -0.37  *P*=.011 | -0.17  *P*=.271 | 1.00 |  |  |  |  |  |  |  |
| **Momentary SC** | -0.36  *P*=.015 | 0.06  *P*=.681 | -0.24  *P*=.106 | -0.13  *P*=.390 | -0.51  *P*<.001 | -0.43  *P*=.002 | 0.40  *P*=.006 | 1.00 |  |  |  |  |  |  |
| **Overall SC** | 0.04  *P*=.808 | 0.11  *P*=.457 | -0.12  *P*=.421 | -0.01  *P*=.958 | 0.22  *P*=.155 | 0.21  *P*=.173 | -0.13  *P*=.397 | -0.06  *P*=.700 | 1.00 |  |  |  |  |  |
| **Adaptive emotion regulation** | -0.25  *P*=.091 | 0.42  *P*=.004 | -0.10  *P*=.506 | 0.03  *P*=.831 | -0.07  *P*=.641 | 0.02  *P*=.899 | 0.09  *P*=.578 | 0.25  *P*=.099 | 0.30  *P*=.047 | 1.00 |  |  |  |  |
| **Maladaptive emotion regulation** | 0.06  *P*=.718 | -0.06  *P*=.681 | 0.02  *P*=.886 | 0.09  *P*=.553 | 0.33  *P*=.027 | 0.09  *P*=.578 | -0.12  *P*=.427 | 0.03  *P*=.831 | 0.46  *P*=.002 | -0.04  *P*=.768 | 1.00 |  |  |  |
| **Training frequency** | -0.03  *P*=.864 | 0.06  *P*=.680 | -0.13  *P*=.384 | -0.12  *P*=.432 | 0.04  *P*=.819 | 0.00  *P*=.993 | -0.03  *P*=.847 | -0.22  *P*=.145 | 0.14  *P*=.378 | -0.13  *P*=.385 | 0.17  *P*=.263 | 1.00 |  |  |
| **WAIP** | 0.27  *P*=.078 | 0.07  *P*=.667 | 0.09  *P*=.556 | 0.14  *P*=.368 | 0.40  *P*=.007 | 0.28  *P*=.069 | -0.16  *P*=.290 | -0.24  *P*=.111 | -0.03  *P*=.838 | -0.07  *P*=.652 | 0.16  *P*=.293 | 0.08  *P*=.622 | 1.00 |  |
| **WAIT** | 0.14  *P*=.385 | 0.06  *P*=.695 | 0.25  *P*=.102 | 0.18  *P*=.257 | 0.07  *P*=.673 | -0.08  *P*=.606 | 0.10  *P*=.522 | -0.10  *P*=.505 | -0.24  *P*=.123 | -0.15  *P*=.351 | 0.06  *P*=.702 | 0.13  *P*=.420 | 0.46  *P*=.002 | 1.00 |

^a^ K10 = Psychological distress at baseline.

^b^ BSI= General psychopathology at baseline.

^c^ SOFAS = Level of functioning at baseline.

^d^ SC = Self-compassion at baseline.

^e^ WAIP= Patient ratings of working alliance.

^f^ WAIT = Therapist ratings of working alliance.
