# Supplementary material for: A Hybrid Ecological Momentary Compassion–Focused Intervention for Enhancing Resilience in Help-Seeking Young People: Prospective Study of Baseline Characteristics in the EMIcompass Trial
Source: JMIR Form Res. 2022 Nov 4;6(11):e39511. doi: 10.2196/39511 (PMC9675017; doi:10.2196/39511)
Supplement: Multimedia Appendix 6 [file formative_v6i11e39511_app6.docx]

# Multimedia Appendix 6 – Variance Inflation Factors and Tolerance

|  | | **Putative mechanisms and processes of change** | | | | | | | |
| --- | --- | --- | --- | --- | --- | --- | --- | --- | --- |
|  | | **Change in overall self-rated**  **self-compassion** | | **Change in momentary self-compassion** | | **Change in adaptive**  **emotion regulation** | | **Change in maladaptive**  **emotion regulation** | |
|  | | VIF ^a^ | Tolerance | VIF | Tolerance | VIF | Tolerance | VIF | Tolerance |
| **Age** | | 1.09 | 0.92 | 1.11 | 0.90 | 1.09 | 0.92 | 1.09 | 0.92 |
| **Gender** | | 1.22 | 0.82 | 1.16 | 0.86 | 1.22 | 0.82 | 1.22 | 0.82 |
| **Ethnic minority status** | | 1.07 | 0.94 | 1.12 | 0.89 | 1.07 | 0.94 | 1.07 | 0.94 |
| **Clinical stage** ^b^ | |  |  |  |  |  |  |  |  |
|  | stage 1b | 1.11 | 0.90 | 1.15 | 0.87 | 1.11 | 0.90 | 1.11 | 0.90 |
|  | stage 2 | 1.44 | 0.69 | 1.48 | 0.67 | 1.44 | 0.69 | 1.44 | 0.69 |
| **Psychological distress** | | 3.35 | 0.30 | 3.33 | 0.30 | 3.35 | 0.30 | 3.35 | 0.30 |
| **General psychopathology** | | 2.96 | 0.34 | 2.92 | 0.34 | 2.96 | 0.34 | 2.96 | 0.34 |
| **Level of functioning** | | 1.26 | 0.79 | 1.33 | 0.75 | 1.26 | 0.79 | 1.26 | 0.79 |

**Table S3.** Variance Inflation Factors / Tolerance for the results displayed in table 3.

^a^ = Variance Inflation Factor.

**Table S4.** Variance Inflation Factors / Tolerance for the results displayed in table 4.

|  | | **Putative mechanisms and processes of change** | | | | | |
| --- | --- | --- | --- | --- | --- | --- | --- |
|  | | **Working alliance – patient rating** | | **Working alliance – therapist rating** | | **Training frequency** | |
|  | | VIF^a^ | Tolerance | VIF | Tolerance | VIF | Tolerance |
| **Age** | | 1.10 | 0.91 | 1.11 | 0.90 | 1.14 | 0.87 |
| **Gender** | | 1.23 | 0.81 | 1.17 | 0.86 | 1.18 | 0.84 |
| **Ethnic minority status** | | 1.08 | 0.92 | 1.09 | 0.92 | 1.15 | 0.87 |
| **Clinical stage** ^b^ | |  |  |  |  |  |  |
|  | stage 1b | 1.17 | 0.86 | 1.16 | 0.86 | 1.15 | 0.87 |
|  | stage 2 | 1.79 | 0.56 | 1.65 | 0.61 | 1.42 | 0.70 |
| **Psychological distress** | | 3.90 | 0.26 | 3.50 | 0.29 | 3.30 | 0.30 |
| **General psychopathology** | | 3.26 | 0.31 | 3.06 | 0.33 | 3.04 | 0.33 |
| **Level of functioning** | | 1.34 | 0.74 | 1.33 | 0.75 | 1.26 | 0.80 |

^a^ = Variance Inflation Factor.

**Table S5.** Variance Inflation Factors / Tolerance for the results displayed in tables 5 and 6.

|  | **Psychological distress** | | **General psychopathology** | |
| --- | --- | --- | --- | --- |
|  | VIF^a^ | Tolerance | VIF | Tolerance |
| **Time** | 1.00 | 1.00 | 1.00 | 1.00 |
| **Age** | 1.45 | 0.69 | 1.45 | 0.69 |
| **Gender** | 1.19 | 0.84 | 1.19 | 0.84 |
| **Ethnic minority status** | 1.28 | 0.78 | 1.28 | 0.78 |
| **Clinical stage** | 1.37 | 0.73 | 1.37 | 0.73 |
| **General psychopathology at baseline** | 4.45 | 0.22 | 4.45 | 0.22 |
| **Level of functioning at baseline** | 1.38 | 0.72 | 1.38 | 0.72 |
| **Overall self-rated self-compassion at baseline** | 2.01 | 0.50 | 2.01 | 0.50 |
| **Momentary self-compassion at baseline** | 1.89 | 0.53 | 1.89 | 0.53 |
| **Adaptive emotion regulation at baseline** | 1.80 | 0.56 | 1.80 | 0.56 |
| **Maladaptive emotion regulation at baseline** | 2.19 | 0.46 | 2.19 | 0.46 |

^a^ = Variance Inflation Factor.
