# Supplementary material for: A Hybrid Ecological Momentary Compassion–Focused Intervention for Enhancing Resilience in Help-Seeking Young People: Prospective Study of Baseline Characteristics in the EMIcompass Trial
Source: JMIR Form Res. 2022 Nov 4;6(11):e39511. doi: 10.2196/39511 (PMC9675017; doi:10.2196/39511)
Supplement: Multimedia Appendix 7 [file formative_v6i11e39511_app7.docx]

# Multimedia Appendix 7 – Cross differences

**Method**

We computed cross differences to compare developments in psychological distress and general psychopathology from post-intervention to follow-up for different levels of baseline characteristics. Cross differences can be understood as the difference of differences [1].

**Results**

Table S displays the cross differences for psychological distress and general psychopathology.

**Table S6.** Cross differences.

|  | | **Cross differences in psychological distress** | **Cross differences in general psychopathology** |
| --- | --- | --- | --- |
| **Age** | | | |
|  | Low vs. high | 2.82 | 0.10 |
| **Gender** | | | |
|  | Female vs. Male | -4.94 | -3.81 |
| **Ethnic minority status** | | | |
|  | White majority vs. minority | -2.99 | 3.01 |
| **Clinical stage** ^e^ | | | |
|  | Stage 1a vs. stage 1b | 0.04 | -1.94 |
|  | Stage 1a vs. stage 2 | 2.38 | -0.70 |
| **General psychopathology at baseline** | | | |
|  | Low vs. high | 6.71 | - |
| **Psychological distress at baseline** | | | |
|  | Low vs. high | - | -4.10 |
| **Level of functioning at baseline** | | | |
|  | Low vs. high | 2.66 | 4.67 |
| **Overall self-rated self-compassion at baseline** | | | |
|  | Low vs. high | 2.06 | 6.96 |
| **Momentary self-compassion at baseline** | | | |
|  | Low vs. high | 4.12 | 7.14 |
| **Adaptive emotion regulation at baseline** | | | |
|  | Low vs. high | -1.66 | -7.59 |
| **Maladaptive emotion regulation at baseline** | | | |
|  | Low vs. high | 4.16 | 0.52 |

*Psychological distress*

Comparing post-intervention and follow-up assessments of psychological distress, we observed positive cross differences for younger vs. older participants and negative cross differences for females vs. males and majority vs. minority participants. This descriptively indicates that being older, male and from an ethnic minority group was associated with more positive developments from post-intervention to follow-up. Regarding clinical characteristics, we observed positive cross differences for stage 1a vs. stage 2 participants und low vs. high general psychopathology. Descriptively, this indicates a less severe clinical stage and lower level of general psychopathology were associated with more positive developments from post-intervention to follow-up. For level of functioning, however, we observed opposing cross differences, suggesting that a lower level of functioning was associated with more positive developments from post-intervention to follow-up. In addition, we observed positive cross differences for baseline levels of self-compassion and maladaptive emotion regulation and a negative cross difference for adaptive emotion regulation. This descriptively indicates that low overall and momentary self-compassion, low levels of maladaptive and high levels of adaptive emotion regulation associated with more positive developments from post-intervention to follow-up.

*General psychopathology*

Comparing post-intervention and follow-up assessments of, we observe a negative cross difference for gender, a positive cross difference for ethnic minority status and no large values for age. This descriptively indicates that developments between post-intervention and follow-up did not differ between older and younger participants, whereas being male and from the ethnic majority was associated with more positive developments from post-intervention to follow-up. For clinical characteristics, we descriptively observed more positive developments from post-intervention to follow-up for participants from stage 1b vs. stage 1a and stage 2 vs. 1a, participants with higher level of psychological distress at baseline, and lower levels of functioning. In addition, we observed positive cross differences for baseline levels of self-compassion and maladaptive emotion regulation and a negative cross difference for adaptive emotion regulation. This descriptively indicates that low overall and momentary self-compassion, low levels of maladaptive and high levels of adaptive emotion regulation associated with more positive developments from post-intervention to follow-up.

**References**

1. Puhani PA. The treatment effect, the cross difference, and the interaction term in nonlinear “difference-in-differences” models. Economics Letters. 2012 2012/04/01/;115(1):85-7. doi: <https://doi.org/10.1016/j.econlet.2011.11.025>.
